# Hyper-Sensitivity to Pitch and Poorer Prosody Processing in Adults With Autism: An ERP Study

**DOI:** 10.3389/fpsyt.2022.844830

**Published:** 2022-05-25

**Authors:** Sarah M. Haigh, Pat Brosseau, Shaun M. Eack, David I. Leitman, Dean F. Salisbury, Marlene Behrmann

**Affiliations:** ^1^Department of Psychology and Institute for Neuroscience, University of Nevada, Reno, NV, United States; ^2^Department of Psychology, Carnegie Mellon University, Pittsburgh, PA, United States; ^3^School of Social Work, University of Pittsburgh, Pittsburgh, PA, United States; ^4^Division of Translational Research, National Institute of Mental Health, Bethesda, MD, United States; ^5^Department of Psychiatry, University of Pittsburgh School of Medicine, Pittsburgh, PA, United States; ^6^Neuroscience Institute, Carnegie Mellon University, Pittsburgh, PA, United States

**Keywords:** auditory, pitch, prosody, ERP, emotion

## Abstract

Individuals with autism typically experience a range of symptoms, including abnormal sensory sensitivities. However, there are conflicting reports on the sensory profiles that characterize the sensory experience in autism that often depend on the type of stimulus. Here, we examine early auditory processing to simple changes in pitch and later auditory processing of more complex emotional utterances. We measured electroencephalography in 24 adults with autism and 28 controls. First, tones (1046.5Hz/C6, 1108.7Hz/C#6, or 1244.5Hz/D#6) were repeated three times or nine times before the pitch changed. Second, utterances of delight or frustration were repeated three or six times before the emotion changed. In response to the simple pitched tones, the autism group exhibited larger mismatch negativity (MMN) after nine standards compared to controls and produced greater trial-to-trial variability (TTV). In response to the prosodic utterances, the autism group showed smaller P3 responses when delight changed to frustration compared to controls. There was no significant correlation between ERPs to pitch and ERPs to prosody. Together, this suggests that early auditory processing is hyper-sensitive in autism whereas later processing of prosodic information is hypo-sensitive. The impact the different sensory profiles have on perceptual experience in autism may be key to identifying behavioral treatments to reduce symptoms.

## Introduction

Autism is associated with a range of social, communication, and sensory symptoms (1) making it difficult to identify mechanisms that can account for all aspects of the complex phenotype. Within the sensory domain alone, there are seemingly conflicting reports of increased sensitivity (over-responding) to sensory information, for example, being over-whelmed in a social environment, while there are also reports of decreased sensitivity (under-responding), for example, missing emotional intent during a conversation. Recently, there has been a growing interest concerning how these seemingly independent sensory symptoms may relate to one another. Very few studies measure sensory responses to different types of sensory stimuli in the same individuals making it difficult to examine how different aspects of autism behavior can manifest simultaneously [as recommended by (2); for example, when measuring the effects of stimulus complexity on the temporal binding window, (3, 4); speech, (5); or word complexity, (6)]. Therefore, in the current study, we examined sensory processing to simple auditory stimuli (that differ in their pitch) that are processed early in the auditory system and processing to stimuli (that differ in their emotion) that are processed later in the auditory system in the same individuals with autism to examine the co-occurrence of different sensory profiles.

### Sensory Profiles of Early Auditory Processing in Autism

Examination of early auditory responses to simple stimuli reveals that the characteristics of the sensory abnormalities range from increased sensitivity and hyper-responsiveness to reduced sensitivity and hypo-responsiveness [for review, see (7)]. There are several ERP measures that can be used to indicate auditory sensitivity: mismatch negativity (MMN) is a preconscious ERP that appears in response to the detection of a deviant stimulus (8). The P3 response on the other hand, reflects early implicit attention to a deviant stimulus and is more commonly elicited when stimuli take more time to process (5, 9). One of the more consistent findings of *hypo*-responsiveness in autism is in the processing of auditory stimuli that vary in their timing. Impaired MMN to duration and frequency-duration deviants were reported, even when P3a to the duration deviant was larger [children: (10)], suggesting early auditory processing of timing is impaired even if later processing (that potentially involves more cognitive mechanisms) is preserved. It is important to note that while the MMN may act as a trigger to the orienting P3 response, there is low correlation between MMN and P3 amplitudes, suggesting that they are not reflecting the same change detection process (11). Similarly, the object-related negativity (ORN; a negative deflection occurring at around 250 ms after the onset of a group of sounds that are perceived as a single object) to auditory patterns was smaller in autism than controls (12, 13), suggesting impairments in segregating the auditory scene that may impact change detection abilities. Smaller ERPs to click-pairs were found to be related to deficits in language processing (14), and in sensory seeking behaviors (15).

On the other hand, several studies have reported *hyper*-sensitivity. Individuals with autism typically exhibit superior behavioral measures of pitch processing (16) that is more evident in those with greater symptom severity [(17), higher ADOS scores; (6), parental reports of emotional problems, (18)], have poorer linguistic abilities (17, 19, 20), and worse emotion recognition (21). It should be noted that sensory hyper-sensitivity has been linked to over-arousal and increased physiological responses in autism (22), and so the hyper-sensitivity may come at the cost of exacerbating symptoms associated with autism. Electrophysiological markers of pre-attentive deviance detection (MMN; and P3a) to simple pitch deviants have been shown to be larger in children with autism (23) and in adults with autism (24), and show slower attenuation compared to controls [adults; (23, 25)]. The auditory N1 [first negativity in the auditory ERP that reflects the earliest processing in primary auditory cortex; (26)] has also been shown to be more abnormal (smaller N1b) in individuals with greater symptom severity (27). Together, this suggests *hyper*-sensitivity in early auditory processing in autism.

A further issue for consideration is that the abnormal auditory sensitivity may be related to deficits in adaptation to auditory input. Millin and colleagues (25) found smaller responses to pitch deviants but also worse adaptation in adults with autism and suggested that adaptation may contribute to some of the auditory sensitivity. The subcortical frequency-following response also increases with repetition and was found to correlate with behavioral measures of sensory overload in children with autism (28), further supporting the contribution of abnormal adaptation to auditory sensitivity. If adaptation is abnormal in autism, then the differences in the stimulus presentation across studies may also be impacting whether auditory processing appears to be hyper- or hypo-sensitive.

Another issue to consider is that the amplitude of neural responses in autism tends to be more variable from one trial to the next (greater trial-to-trial variability; TTV) compared to controls, which may be impact the ERP amplitudes when averaging across trials. Greater TTV has been reported in auditory responses in the fMRI signal (29–31), and in the visual ERP response (32) but, interestingly, not in electrodermal (EDA) responses to olfactory inputs (33), and so may be modality specific. When TTV and amplitude in sensory signals are compared, they may provide a description of the quality of the incoming signal, with greater TTV suggesting lower fidelity signal.

### Later Auditory Processing in Autism

Auditory processing of more complex auditory stimuli, such as emotion in vocal utterances (prosody) are processed later in the auditory processing stream, and is often worse in autism (21, 34, 35), particularly for speech during noise (36). ERPs to prosodic utterances elicit smaller ERPs [children; (37)], even when simple auditory cues were the deviants within speech [children; (38)], and smaller prosodic ERPs were present in those with greater auditory sensitivity (37). This is likely related to under-activation of the superior temporal sulcus when processing speech (35, 39–41). Lepistö et al. (5) found that children with autism had larger pitch and larger phoneme MMN when the pitch features of the phonemes remained consistent. However, when the phonemes were more speech-like and varied in pitch, MMN was smaller in autism. Together, these findings highlight the need to characterize sensory responses to simple and complex stimuli and along the sensory processing stream to be able to begin identifying how these sensory profiles might interact.

In the current study, we conducted two studies to examine ERP responses to (a) changes in simple pitch stimuli and (b) changes to more complex prosodic utterances using a roving MMN paradigm (41). The aim was to be able to characterize the responses to these two different tasks to identify if seemingly different sensory profiles manifest in the same individuals under similar experimental conditions. We chose to investigate sensory processing in an adult population as autism is a lifelong diagnosis, and to ensure that any effects of autism were not merely due to differences in developmental trajectory.

To separate the potential effects of abnormal pitch/prosody processing from abnormal adaptation (similar to those reported by 24), we manipulated the number of sounds presented before the pitch or emotion changed, with the assumption that if adaptation is abnormal in autism then this should be evident after more sounds are presented. If autism is associated with hyper-sensitivity to sounds, then the individuals with autism should show abnormal EEG responses to any change in sound. However, if the hyper-sensitivity in autism is associated with abnormal adaptation, then EEG responses will only differ when comparing the short vs. long sound trains in autism as the more presentations of a sound, the greater the release from adaptation after the sound changes (42–44).

To examine the early sensory profile, the amplitude of N1 and MMN ERP responses to changes in pitch, as well as the TTV in the waveform to provide an index of signal quality, were compared between the autism and control groups. We focused on N1 and MMN as they are early pre-attentive responses of sensory processing and are larger when a deviant sound is detected (45–47). However, while N1 reflects the response to the individual tone, MMN is a subtraction waveform that isolates the response to the deviant tone relative to the response to the previous (standard) tones and, hence, is sensitive to deviance detection (8). MMN has been shown to be sensitive to diagnosis, being reliably reduced in conditions such as schizophrenia (48) and dyslexia [for a review, see (49)]. There is also some evidence that the generators of the N1 and MMN differ slight, where N1 localizes to primary auditory cortex and the MMN just outside, possibly due to some prefrontal involvement in identifying the deviant sound (50). Because the MMN to changes in pitch can be impacted by the deviant and standard N1 responses due to their appearance around the same time, we analyzed the effects of pitch on deviant and standard N1s separately to identify the component/s driving any effects on the MMN. In addition, to assess whether any differences in MMN were due to abnormal auditory segmentation when grouping the repeated standard tones, we analyzed the slow-wave potentials to the standard tones [similar to (51, 52)].

To examine the sensory profile to more complex stimuli that are processed later in the processing steam, we focused on the P3 response to changes in vocal utterances that differed in their emotion, the slow-wave potentials to the standard utterances, and TTV to examine the stability in responses to prosodic stimuli between groups. We did not assess N1 and MMN responses as change detection to complex stimuli is not always stable early in the ERP response [for example, see (53) where there is no MMN to complex patterns of tones]. The longer presentation duration of the utterances enabled examination of the later P3 response to the change in prosody which was not possible for the tones.

We predicted that adults with autism would exhibit heightened sensitivity to changes in pitch and reduced sensitivity to changes in prosodic stimuli compared to their matched-neurotypical controls. Owing to the N1, MMN, and P3 responses all being typically maximal at frontocentral electrodes, with the N1 and MMN responses typically being maximal in slightly more anterior electrodes than the P3 (5, 8, 54), we focused our investigation at these electrodes to avoid multiple comparisons across all possible electrode locations (53). We then conducted exploratory analyses to see if there was a relationship between responses to pitch and responses to prosody to identify if responses to one type of sensory stimulation was related to the other.

## Experiment 1—Pitch Processing

### Materials and Methods

#### Participants

Twenty-four adults with autism (19 males, 5 females; mean age: 28.5 years, SD: 7.4 years, range: 19–44) and 28 neurotypical controls (19 males, 9 females; mean age: 28.7 years, SD: 8.1 years; range: 19–47) matched for age and gender participated (see Table 1 for demographic and symptom score distributions). Computing a post-hoc sensitivity analysis [G^*^Power; (55)] from a mixed-measures ANOVA, estimating 80% power using this sample size (*N* = 52), generated a large effect size (Cohen's *d* = 1.0). A medium effect size in ERP responses between autism and controls is consistent with previous findings in this area and the sample size used here is large compared to previous studies [Lepistö et al. (5) = 15 children; Ludlow et al. (37) = 11 children; Kujala et al. (24) = 8 adults; Millin et al. (25) = 18 adults], and so the current sample is large enough to detect meaningful differences in sensory ERP responses between autism and control groups. None of the participants reported any recent significant head injury, were pregnant, or reported any hearing or vision loss. Participants gave their informed consent to take part in the 2-h study and were paid $50 for their time. All protocols were approved by the Institutional Review Board at Carnegie Mellon University.

**Table 1:** Demographic and symptom information for the autism and control groups, showing mean, standard deviation (SD), skewness, and kurtosis for age, IQ, ADOS communication, ADOS reciprocal social interaction, and calibrated symptom severity scores.

	**Autism**	**Controls**
Gender (M/F)	19 / 5	19 / 9
Age (years)	28.54	28.71
SD Age	7.35	8.06
Skew Age	0.58	0.79
Kurtosis Age	2.23	2.50
IQ	111.71	
SD IQ	14.94	
Skew IQ	−0.28	
Kurtosis IQ	1.60	
ADOS Communication	3.58	
SD Communication	1.35	
Skew Communication	1.19	
Kurtosis Communication	5.39	
ADOS Social	6.63	
SD Social	2.00	
Skew Social	0.49	
Kurtosis Social	2.15	
Symptom Severity	5.46	
SD Severity	1.85	
Skew Severity	0.18	
Kurtosis Severity	1.90	

The individuals with autism all met DSM-IV or DSM-V criteria for autism and had full IQ scores above 85. Clinical diagnosis was confirmed with the Autism Diagnostic Observation Schedule (ADOS module 4) (56) and by assessment carried out by expert clinicians at the Center For Excellence in Autism Research at the University of Pittsburgh. Total ADOS scores were also converted into calibrated symptom severity scores [(57); see Supplementary Table S1 for demographic and diagnostic information]. A study comparing the DSM-IV and the DSM-V criteria showed that the participants who met the criteria for autism under the DSM-IV also met the criteria for autism under the DSM-V (58).

The neurotypical controls were students from Carnegie Mellon University and the surrounding Pittsburgh area. None of the controls had a neurological or psychiatric diagnosis and were not taking any medications at the time of the experiment.

#### Stimuli

All of the tones were generated in MATLAB and presented using the PsychToolbox extension (59–61). Each frequency tone was presented for 50 ms and included a 5 ms ramp up and ramp down to avoid high frequency artifacts from the earphones. Tones were sampled at 48 kHz with 16-bit resolution.

#### Stimuli for Measuring EEG Responses to Pitch

**Figure 1:**
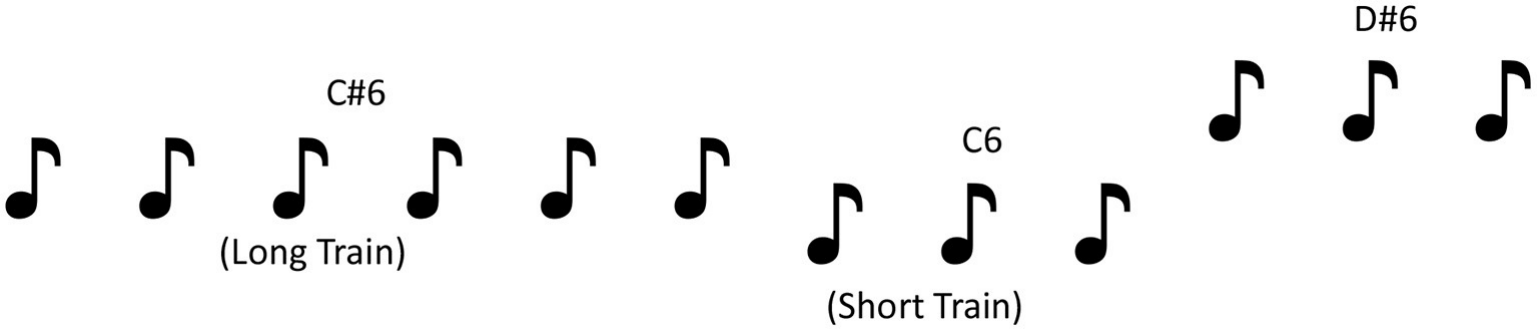
Illustration of the roving pitch paradigm used in the EEG task.

Stimuli were 1046.5 Hz (C6), 1108.73 Hz (C#6), and 1244.51 Hz (D#6) tones. The three temporal frequencies provided three pitch changes: small (C6–C#6), mid (C#6–D#6), and large (C6–D#6). Each tone was presented either three or nine times consecutively with a 330 ms inter-stimulus interval (equivalent of 3Hz) before changing to another pitch tone, generating a roving paradigm (40; see Figure 1 for illustration). Roving paradigms have the added benefit over standard MMN paradigms by ensuring that all deviants become standards, eliminating the effects of stimulus-specific effects, while offering the opportunity to manipulate and measure the effects of adaptation (42–44) on MMN measures. The order of pitch presentation (C6, C#6, or D#6) and length or train (three or nine tones) was pseudo-randomized so that pitch always changed at the end of the train.

#### EEG Data Acquisition

A 64-channel BioSemi Active2 EEG system (Amsterdam, Netherlands) using a standard montage was used to collect the data. Electrodes were held in place using a nylon head cap. Four additional electrodes were placed around the eyes to monitor eye movements: one above the right eye, one below the right eye, and on the outer canthi of each eye. Two electrodes were added to the mastoids. One electrode was attached to the collarbone to detect heartbeat. All electrodes were recorded relative to the standard BioSemi CMS and DRL electrodes. Data were digitized at 512 Hz with a 24-bit A/D conversion.

#### Procedure

Tones were presented every 330ms and were either presented three or nine times before changing pitch. Participants were instructed to ignore the tones and keep their eyes on the fixation cross to reduce ocular artifact. To ensure that the participants were still engaged and attending to the fixation cross, an attention manipulation was included: for 16.6% of tone trials, the central fixation cross flashed white for 100 ms before returning to black. Participants were asked to replace the spacebar whenever they saw the fixation cross flash white. Directing the participants not to attend to the tones ensured that any fluctuations in attention were less likely to impact the ERP signal.

To verify that all participants were able to distinguish perceptually between different pitched tones, participants completed a brief behavioral pitch discrimination task. Responses showed that both groups had similar pitch discrimination functions, although the autism group were slower at responding (see Supplementary Material for more information).

#### Data Analysis

EEG data were preprocessed and analyzed using MATLAB (MathWorks) and the EEGLAB (62) and ERPLAB (63) toolboxes. EEG data were re-referenced offline to the average of the mastoids and filtered using a 0.1–100 Hz Butterworth zero-phase filter. The data were checked visually for noisy channels (high amplitude signal that differed from the remaining channels or signal that was completely flat due to resting on too much hair). Any noisy channels were interpolated (0.8% of electrodes from the autism group and 0.5% of electrodes from the neurotypical controls). An independent component analysis (ICA) was then used to manually identify components that contained blinks, horizontal eye movements, and heartbeat, which were then removed from the dataset. The data were then epoched relative to the onset of the sounds.

For the roving tone paradigm, the epochs from 50 ms before the onset of the tone to 330 ms after the onset of the tone were extracted and baseline corrected between −50–0 ms before the onset of the tone. Any epochs that contained signal ±100 μV were automatically rejected from analysis. All epochs were then filtered with a low-pass 20 Hz filter. Epochs from the beginning and end of each train were averaged for each stimulus type (for example, the first tone of the long C#6 train, the last tone of the long C#6 train, the first tone of the short C#6 train, the last tone of the short C#6 train etc.). The waveform from the end of the tone trains became the “standard” and the waveform from the beginning of the tone trains became the “deviant”. The N1 from the deviant waveform, the N1 from the standard waveform, and the MMN from the subtraction waveform were calculated by averaging the response 110–130 ms after stimulus-onset. The standard waveform was subtracted from the deviant waveform to visualize MMN.

The responses to the standard tones were assessed for ERP markers of auditory segmentation. The slow-wave potential generated over the course of the three standard tone train and the nine standard tone train were analyzed. For the slow-wave analysis only, the data were low-pass filtered at 1.5 Hz consistent with previous measures of auditory scene analysis (51, 52). For the short tone train, epochs −100–1,000 ms were extracted and for the long tone train, epochs −100–3,000 ms were extracted. To capture the majority of the negative deflection in the response, the average signal from 250 to 750 ms was calculated for the short tone train and 250–2,750 ms for the long tone train.

**Figure 2:**
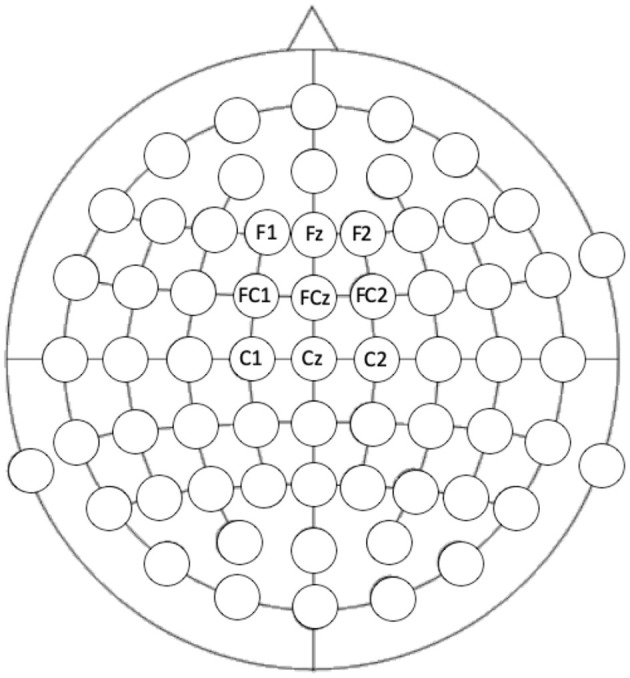
Electrode locations that were extracted for analysis. Location of the electrode was included in the analysis: frontal, frontocentral, and central rows compared to the left, central, and right columns.

Auditory ERPs, specifically auditory N1 and MMN are maximal at frontocentral locations (64) and so responses at electrodes F1, Fz, F2, FC1, FCz, FC2, C2, Cz, and C2 were extracted (see Figure 2 for locations). Including electrodes either side of the midline ensured that any hemispheric differences would also be captured. Electrode location was included as a variable in the analyses. Mixed-model ANOVAs were conducted to assess the effect of the length of the train (short vs. long), electrode column (electrodes covering right hemisphere x central x left hemisphere), electrode row (frontal x frontocentral x central electrode chains) x group (autism x control) on the deviant N1, the standard N1, the MMN (standard-deviant N1), and the slow-wave potential to the standard tone trains separately.

Similar mixed-model ANOVAs were then used to assess the effect of pitch difference (small x medium x large), row, column, and group on N1 and MMN responses. The effect of the length of tone train and of the effect of pitch difference on ERPs were analyzed separately to ensure that the maximum number of epochs were used to calculate each individual's average waveform, making the signals as reliable as possible.

To assess TTV, the standard error of the mean (SEM) was calculated for each participant for each data point in the epoch. SEM was calculated for each data point between 110 ms (the beginning of the N1) to the end of the epoch (330 ms), and then the average SEM was calculated for each participant, for each condition, for each channel, and analyzed using a separate mixed-measures ANOVA, similar to the analysis of the peak amplitude.

Significant interactions in all ANOVAs were explored using post-hoc *t*-tests and Cohen's *d* effect sizes. The degrees of freedom were corrected if the assumption of heterogeneity was violated to generate a more appropriate *p*-value.

To ensure that all participants were awake and were not overly fatigued during the EEG recording, responses to the change in fixation cross color were analyzed. Reaction times >5 s were removed from analysis and were assumed to be missed targets. The median reaction time to the remaining targets was then calculated for each participant. The ratio of missed compared to the total number targets presented was also calculated. To identify if there was a difference between the autism and control groups in their responses to the fixation cross, independent samples *t*-tests were used.

To assess the relationship between ERPs to pitch processing and ADOS, symptom severity, and IQ scores, Spearman's correlations were calculated. We chose non-parametric correlations owing to the heavy skew and kurtosis in the demographic and symptom data (see Table 1). To reduce the number of correlations being conducted, scores were only correlated with the responses from the electrode that produced the largest ERP amplitude for both groups (F2; although the same pattern of correlations was found in Fz). In addition, only conditions that produced significant group differences were analyzed. This method reduced the number of correlations conducted while also focusing on comparisons where any relationship with symptoms would have been expected. No multiple comparisons correction was added and so these analyses are exploratory.

The MATLAB scripts for stimulus presentation and data analysis are on GitHub (SarahMHaigh/AuditoryInAutism) and data are available through the Open Science Framework (https://osf.io/pnvay/).

### Results

To identify differences in pitch processing between adults with autism and their matched neurotypical controls, we first compared the autism and control groups on their ERPs to changes in pitch and compared the amplitude of the MMN and N1, and the trial-to-trial variability in ERP signal, between the autism and control groups. We then compared the ERPs to symptom measures in the autism group, to identify whether those who are more symptomatic showed greater abnormalities in pitch processing.

#### ERPs to Roving Pitch

We assessed the MMN and N1 responses to the roving pitch paradigm by first focusing on the effect of tone train length (3 or 9 tones before a pitch change), and second, on the effect of the change in the pitch (large, mid, or small). We compared the effects of tone length, electrode location (row and column, see Figure 2 for head model), and group (autism and control) on MMN and N1 response amplitudes to the deviant and standard tones separately.

For the MMN responses, there was no significant difference between the short and long tone trains [*F* (1, 50) = 0.12, *p* = 0.736], however, there was a significant interaction between length x row [*F* (2, 100) = 3.16, *p* = 0.047] due to the long train evoking a nominally larger MMN in the frontal electrode row compared to the other electrode rows (not significant in post-hoc analyses; *d* < 0.13). The waveforms for each electrode location to the short and long tone trains for the autism and control groups are shown in Supplementary Figures S2–S4.

**Figure 3:**
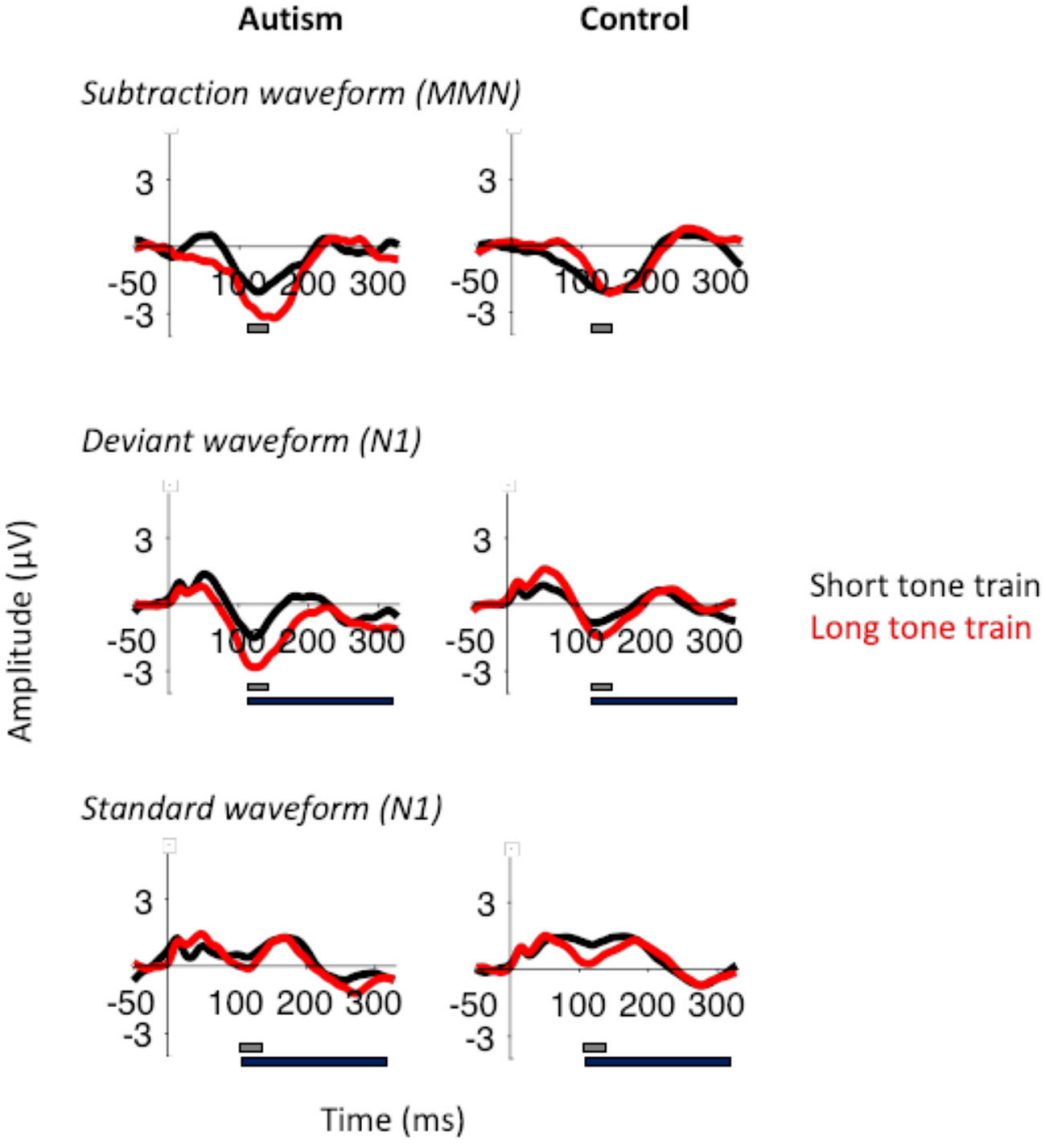
MMN (top), deviant N1 (middle), and standard N1 (bottom) responses after the short tone train (black) and long tone train (red) for autism (left) and neurotypical controls (right). The gray bar indicates the window used for the peak amplitude analysis. The black bar indicates the window used for the TTV analysis. MMN was significantly larger after the long tone train in autism.

**Figure 4:**
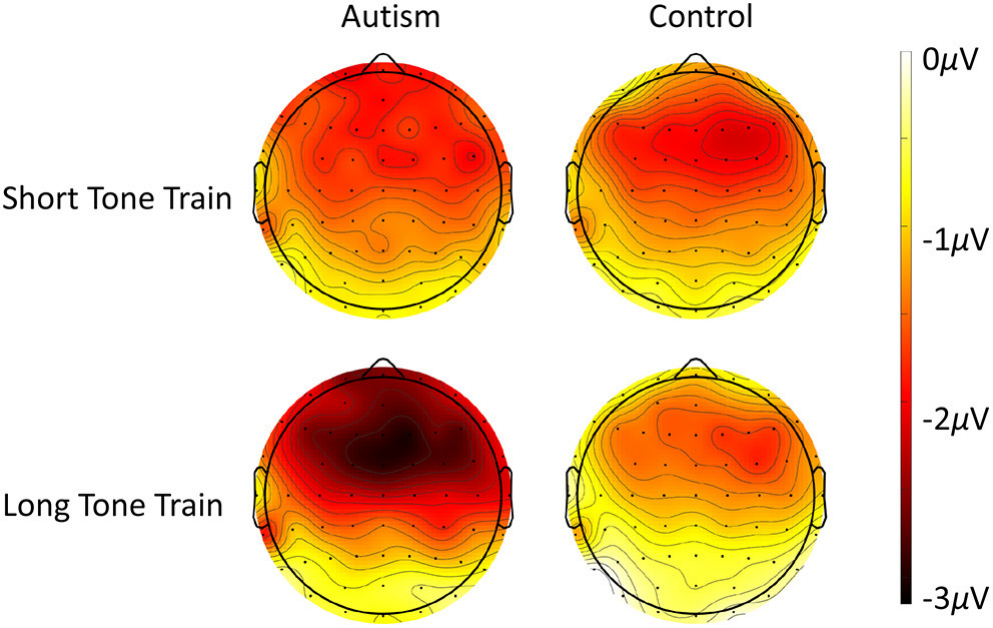
Scalp topography of the MMN response to the deviant after the short tone train (top row) and the long tone train (bottom row) for autism (left column) and neurotypical controls (right column). Note that negative (darker color) reveals site of highest deviant response, as evident especially over frontal areas to the long tone train trials.

When comparing the autism and control groups, MMN was not significantly different [*F* (1, 50) = 1.53, *p* = 0.222], but there was a significant interaction between length x group x column [*F* (2, 100) = 3.25, *p* = 0.043; Figure 3] where, compared with the control group, the autism group exhibited a larger MMN after the change from the long tone train compared to the short tone train and this effect was strongest over the right hemisphere [*t* (130.4) = 3.27, *p* = 0.001; *d* = 0.53; See Supplementary Table S2 for means and SDs for this interaction]. There was no significant interaction between group and row. Topography of MMN activity (Figure 4) shows similar patterns of activation across the scalp in autism and controls with greater amplitudes in frontal areas, that is slightly stronger over the right hemisphere.

To identify what was driving the MMN differences, we assessed N1 responses to the deviant and standard tones that were used to generate the MMN. For the N1 responses to the deviant tones, there was no significant difference between the groups overall [*F* (1, 50) = 2.3, *p* = 0.136], and there was a marginal difference between length x row [*F* (2, 100) = 2.90, *p* = 0.060], which is similar to MMN responses. However, the deviant N1 response was significantly larger after the long tone train [*F* (1, 50) = 7.05, *p* = 0.011], and the interaction between length x group x column was not significant [*F* (2, 100) = 0.59, *p* = 0.555].

For the N1 responses to the standard tones, there was similarly no significant difference between the groups, [*F* (1, 50) = 0.42, *p* = 0.519], and a marginal increase in the standard N1 response to the long tone train [*F* (1, 50) = 3.54, *p* = 0.066], similar to the deviant N1 response. Again, there was a significant interaction between length x row [*F* (2, 100) = 3.90, *p* = 0.023] and a length x group x column interaction [*F* (2, 100) = 3.79, *p* = 0.026]. The interaction with group was due to the control group producing a larger N1 response to the long standard tone train compared to the short standard tone train in all electrodes, but particularly in the midline and right hemisphere electrodes [right: *t* (83) = 3.25, *p* = 0.002; *d* = 0.37; center: *t* (83) = 3.24, *p* = 0.002; *d* = 0.36; left: *t* (83) = 2.90, *p* = 0.005; *d* = 0.32]. Therefore, the effects of tone train length and the differences between autism and control groups in their MMN responses, appear to be primarily drive by differences in adaptation reflected in the standard N1 response.

**Figure 5:**
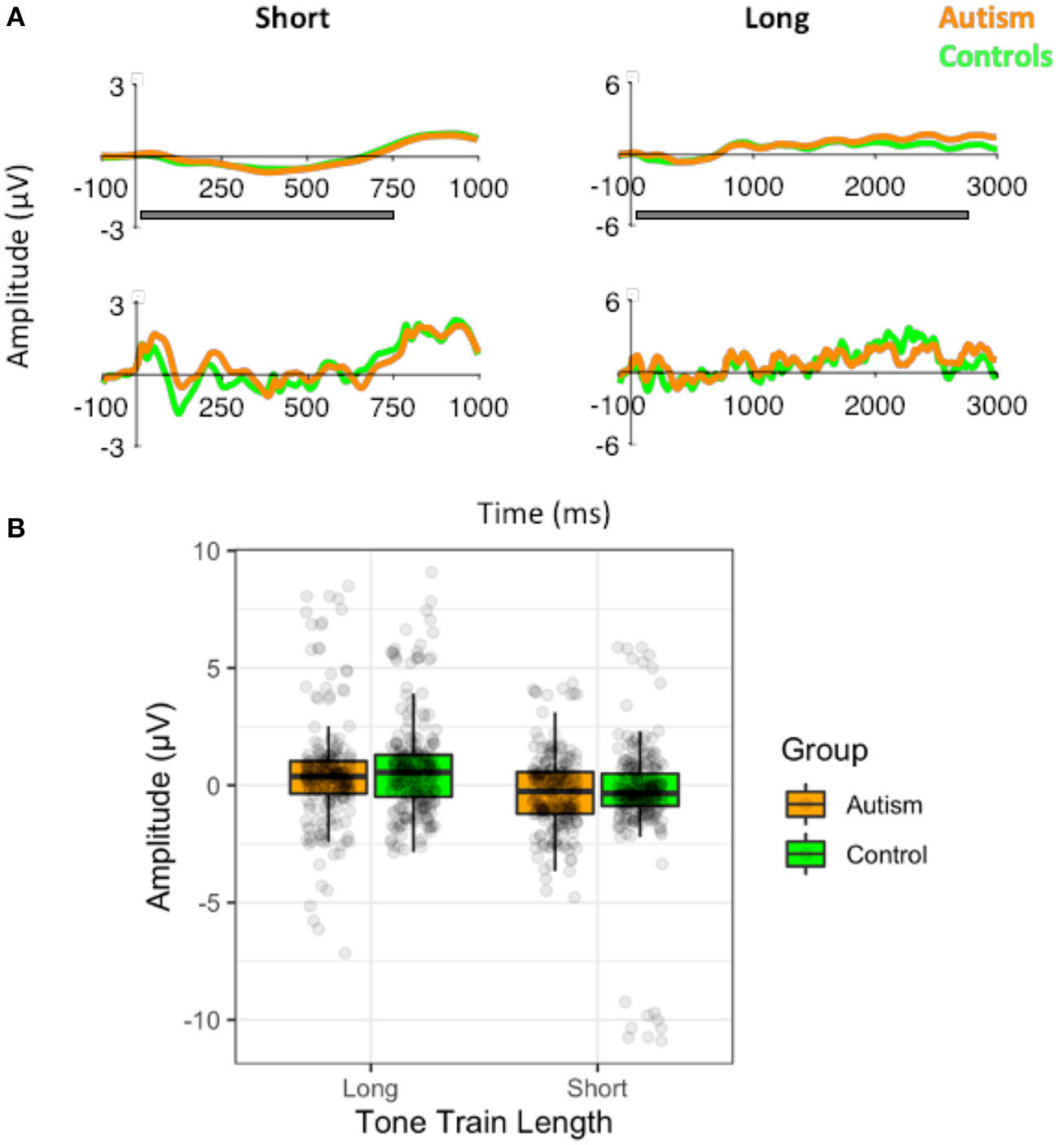
**(A)** Waveforms to the short (3) standard tone trains and the long standard tone trains (10) filtered at 1.5Hz for the analysis of the slow-wave (top) and without the filtering (i.e., the original filtered data for the MMN analysis; bottom), shown separately for individuals with autism and neurotypical controls. Both versions of the filtered waveforms are shown to illustrate the slow-wave that was analyzed and how it arose from the original data used to calculate the MMN. The gray bar indicates the window use for the slow-wave analysis. **(B)** Amplitudes of the slow-waves for the long and the short standard tone trains shown separately for individuals with autism and neurotypical controls.

To identify whether the larger MMN in autism following the long tone train was due to abnormal auditory segmentation when grouping the standard tones, we compared the slow-wave potentials of the autism and control groups to the short (3) tone train and the long (10) tone train, while accounting for electrode location (row and column). Interestingly, the slow potential was larger during the short tone trains [*F* (1, 51) = 8.08, *p* = 0.006], but there was no significant difference between groups [*F* (1, 51) = 0.03, *p* = 0.863; Figure 5] nor any significant interactions with group. The lack of a group difference suggests that the larger MMN in autism is due to the release from adaptation, rather than abnormal segmentation of the standard tone trains.

**Figure 6:**
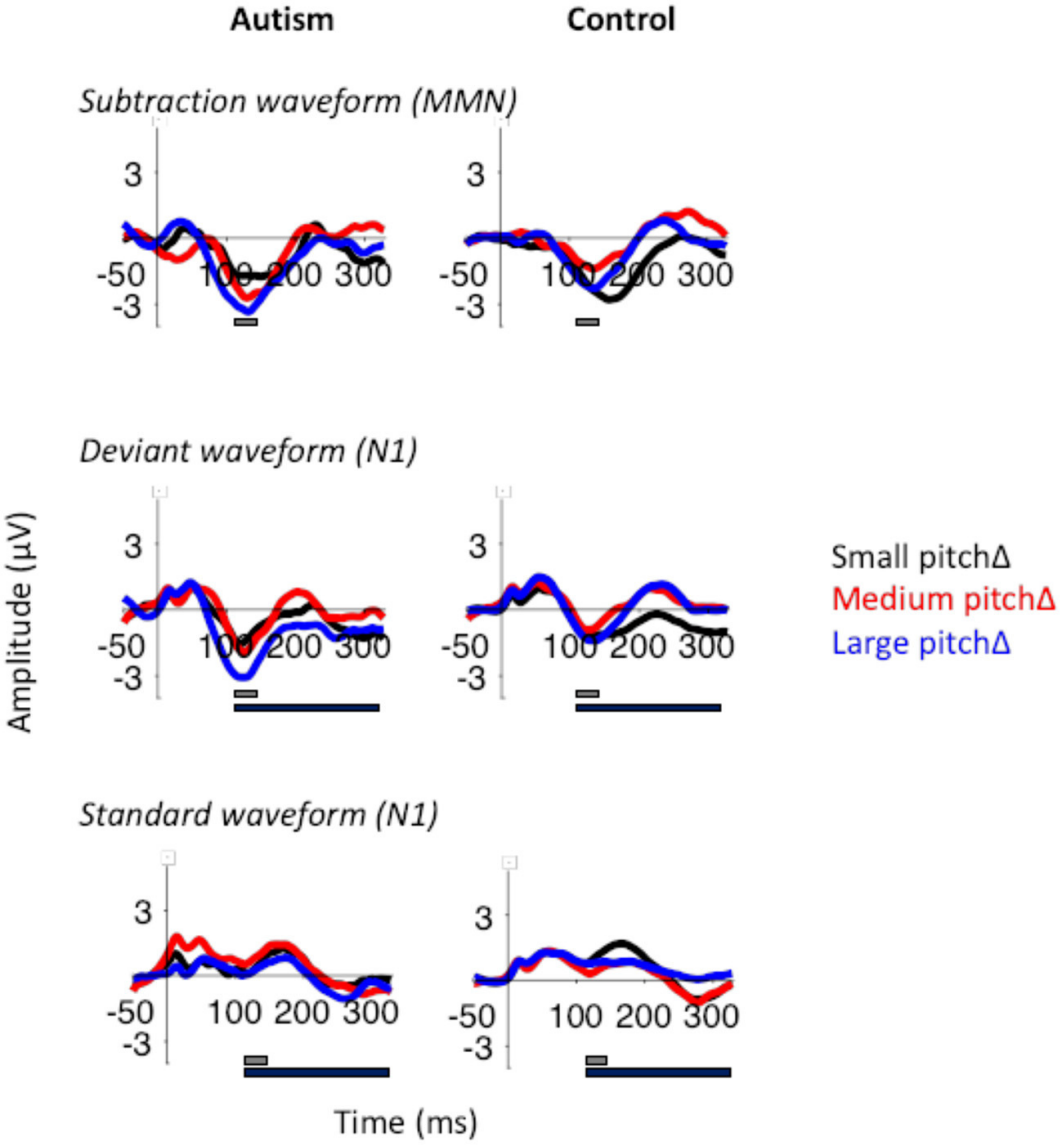
MMN (top), deviant N1 (middle), and standard N1 (bottom) to the small, medium, and large pitch changes (black, red, and blue respectively) in autism (left) and neurotypical controls (right). The gray bar indicates the window used for the peak amplitude analysis. The black bar indicates the window used for the TTV analysis. N1 was larger in response to the large pitch changes, but there were no significant differences between the groups.

When analyzing the effect of change in pitch, there was no significant effect of change in pitch in the MMN [*F* (2, 100) = 1.80, *p* = 0.170] or between groups [*F* (1, 50) = 1.53, *p* = 0.222; Figure 6]. However, the large pitch differences produced the largest deviant N1 response [*F* (2, 100) = 4.62, *p* = 0.012], despite the absence of significant differences between groups in N1 amplitudes [*F* (1, 50) = 2.30, *p* = 0.136]. There were no effects of group or change in pitch in the standard N1 response. For all analyses, MMN and N1 responses were largest in the frontal electrodes (*p* < 0.05; Supplementary Figures S5–S7).

#### Trial-to-Trial Variability

Similar to the analysis of the N1 and MMN amplitudes, we ran a mixed-measures ANOVA comparing electrode location (row and column), and group (autism and control) on TTV.

Analysis of the TTV from the N1 peak to the end of the epoch for responses to all of the tones revealed greater TTV in autism compared to controls [*F* (1, 49) = 4.55, *p* = 0.038; *d* = 0.59]. When just focusing on the responses to the deviant tones, there was only a marginally significant increase in TTV in the autism group [*F* (1, 49) = 3.63, *p* = 0.063; *d* = 0.52]. These effects were greatest in the central column (Fz, FCz, Cz; *p* < 0.05, Bonferroni corrected). There was no significant relationship between TTV in the Fz electrode and the MMN to the large tone train in either group (*p* > 0.05).

#### Reaction Time to Change in Fixation Cross

To ensure that any group differences in ERPs were not due to differences in attention or fatigue, the response to the change in fixation cross color were analyzed. There was no significant difference between the autism and control groups on their reaction times when responding to the fixation target [*t* (48) = 0.93, *p* = 0.356] or in the number of missed trials [where reaction times were >5 s; *t* (42) = 0.68, *p* = 0.500].

#### Relationship Between Pitch Processing and Symptoms

The relationship between ERP responses and symptom scores in the autism group were then assessed. As the MMN response was maximal over F2 and the main effect of autism was evident after the long tone train, the MMN after the long tone trains and the TTV across all epochs in autism were correlated with their ADOS and symptom severity scores. There were no significant relationships between MMN or TTV and ADOS communication or reciprocal social interaction scores, with calibrated symptom severity scores, or with any of IQ scores (*p* > 0.05).

### Interim Discussion

Overall, compared to neurotypical controls, the adults with autism showed larger MMN amplitudes to pitch changes after long tone trains compared to controls, suggesting that abnormal release from adaptation is responsible for auditory sensitivity rather than a specific hyper-sensitivity to pitch (which would have generated a larger MMN in autism after the large pitch difference. These findings are consistent with reference 25. Identifying the behavioral correlates of abnormal releasing from adaptation, for example, on attention switching, could help identify how a large MMN can reflect cognitive symptoms in autism.

One point to note is that the slow-wave was larger during the short (three) compared to the long (nine) tone train. This may have been due to there being at least 3 tones in each standard and so this was more predictable than the 9-tone grouping. However, there were no significant group differences, suggesting that grouping mechanisms are not responsible for the differences in MMN. The greater trial-to-trial variability in autism to all of the tones (and not just to the deviant tones) is consistent with previous findings of greater TTV in visual ERP amplitudes (32) and in fMRI responses in primary auditory cortex (30, 31). The findings presented here replicate and add to the growing evidence that early sensory processing is unstable in autism.

Surprisingly, there were no significant differences between groups in their N1 or MMN amplitudes based on the magnitude of the pitch change. The lack of robust effects of pitch change has been noted before when comparing absolute pitch change to relative pitch change. MMN was more robust to relative pitch change (65), which may be due to the auditory system adapting to the mean pitch; a phenomenon that has been found in category perception to speech (66). Therefore, participants may have adapted to the mean pitch across the roving paradigm.

Next, we assessed prosody processing in the same adults with autism and controls to examine the sensory profile in autism when processing more complex prosodic utterances that are processed later in the processing stream.

## Experiment 2—Processing Prosodic Utterances

### Materials and Methods

#### Participants

The same 24 adults with autism and 28 matched neurotypical controls from experiment 1 participated in experiment 2. Both Experiment 1 and 2 were conducted on the same day in the same experimental session in the same order. Participants were given a break between experiments.

#### Stimuli

Two examples of delight and two examples of frustration from Simon-Thomas et al. (67) were selected. Each example was uttered by a female speaker and by a male speaker. The change in speaker allowed for comparison of changing prosody (delight compared to frustration) to changing sound (delight from speaker 1 to delight from speaker 2 etc.,). This ensured that the P3 response was to the prosody and not the change in speaker. The prosodic utterance was presented three or six times before changing prosody and/or speaker. The length of the long utterance train was shorter than in Experiment 1 due to the fact that the utterances were longer than the 50 ms tones, and shorter train lengths ensured that the experiment did not continue for too long. The same utterance was never presented consecutively. The spectrograms of the utterances are in Supplementary Figure S9.

#### Procedure

For the roving prosody EEG session, utterances were presented every 1.5 s, three or six times, before changing prosody and/or speaker. Similar to the pitch EEG task, participants were instructed to ignore the sounds and focus on the fixation cross. Whenever the fixation cross flashed white (8.3% of trials), they hit the spacebar.

To verify that all participants were able to reliably identify different prosodic utterances and to identify if any ERP differences could be explained by slower identification, participants completed a brief prosody identification task. Responses showed that both groups could equally identify all utterances and had similar reaction times (see Supplementary Material for more information).

#### Data Analysis

For the prosody EEG paradigm, the same analysis method from the roving pitch paradigm was used, except that epochs from 150 ms before the onset of the utterance to 1,500 ms after the onset of the utterance were extracted and baseline corrected between −150–0 ms before the onset of the utterance. Any epochs that contained signal ±200μV were automatically rejected from analysis. This is more lenient than in Experiment 1, due to the P3 being larger in amplitude than the N1 and the epoch duration being three times longer. Having a stricter threshold of ±100μV resulted in substantially more epochs being rejected resulting in an unstable waveform to the utterances. Epochs from the beginning and end of each train were averaged for each stimulus type (for example, the first utterance from the long delight prosody train, the last utterance from the long delight prosody train etc.). The waveform from the end of the utterance trains became the “standard” and the waveform from the beginning of the utterance trains became the “deviant”. The peak of the deviant waveform (250–400 ms after stimulus-onset) was extracted as the P3 response. We focused on the P3, instead of the MMN, first, to be consistent with previous studies of prosodic ERP findings, and second, because prosodic utterances do not have sharp on and off-sets making the timings of the N1 noisy when trying to identify an MMN. The peak of the standard waveform (200–350 ms after stimulus-onset) was extracted to identify if there were effects of the different utterances on ERPs that could explain any differences in P3 responses to the change in prosody or any differences between groups. Note that the nearest positivity to a P3 was earlier in the standard waveform than in the deviant waveform and so is likely a P2 response (54).

Similar to Experiment 1, the slow-wave potential generated over the course of the three standard utterance train and the nine standard utterance train were analyzed. For the analysis of the slow-wave potential only, the data were low-pass filtered at 1.5 Hz, and the short utterance trains were epoched −100–4,000 ms and for the long utterance train at −100–9000 ms. To capture the majority of the negative deflection in the response, the average signal from 250 to 3,750 ms was calculated for the short tone train and 250–8,750 ms for the long tone train.

Mixed-model ANOVAs were conducted to assess the effect of the length of the train (short vs. long), column (electrodes covering right hemisphere x central x left hemisphere), row (frontal x frontocentral x central electrode chains) x group (autism x control) on the P3 response and the slow-wave potential (see Figure 2 for head map of electrode locations). A similar mixed-model ANOVA was then used to assess the effect of change in prosody (delight x frustration), row, column, and group on P3 responses. To investigate group effects for each of the changes in prosody, P3 to delight and P3 to frustration were analyzed in separate mixed-model ANOVAs (group x row x column). Remaining post-hoc analyses were conducted using t-tests and Cohen's *d* effect sizes.

Similar to the analysis of TTV during pitch processing, the standard error of the mean (SEM) was calculated for each participant for each data point in the epoch, and then averaged over from 250 ms (the beginning of the P3) to the end of the epoch (650 ms). This was calculated for each participant, for each condition, for each channel, and analyzed using a separate mixed-measures ANOVA, similar to the analysis of the P3 amplitude.

The behavioral responses to the change in fixation cross color were analyzed to ensure that participants were still awake and attending during the EEG recording. This analysis is particularly important as Experiment 2 always followed Experiment 1 and so comparing behavioral responses will highlight if any group differences in ERPs may have been due to one group being more fatigued than the other. Reaction times longer than 5 s were assumed to be missed targets. The number of missed targets as a ratio of the total number of targets and the median reaction time were calculated. Individuals with autism and controls were compared using independent samples *t*-tests.

To assess possible relationships between the P3 and symptoms (ADOS, symptom severity, and IQ scores), and the P3 and MMN response to the pitch deviant we used Spearman's correlations. As these correlations were exploratory, no multiple comparisons corrections were added. However, to reduce the number of correlations conducted, only P3 responses from the electrode that produced the largest response in the group level analysis, FCz (see Results), were analyzed and only under conditions that evoked significant group differences.

### Results

Similar to the analysis of pitch processing, we compared the autism and control groups' ERPs to changes in prosody and then compared the amplitude of the P3 and the trial-to-trial variability in ERP signal. Second, we identified the relationships between the ERPs to prosody processing to symptom measures in the autism group, to identify whether those who are more symptomatic showed greater abnormalities in prosody processing measures. Finally, the responses to pitch and prosody processing were compared to assess whether the abnormal pitch and prosody processing were related, and whether this relationship was evident in the autism and control groups separately.

#### ERPs to Roving Prosody

**Figure 7:**
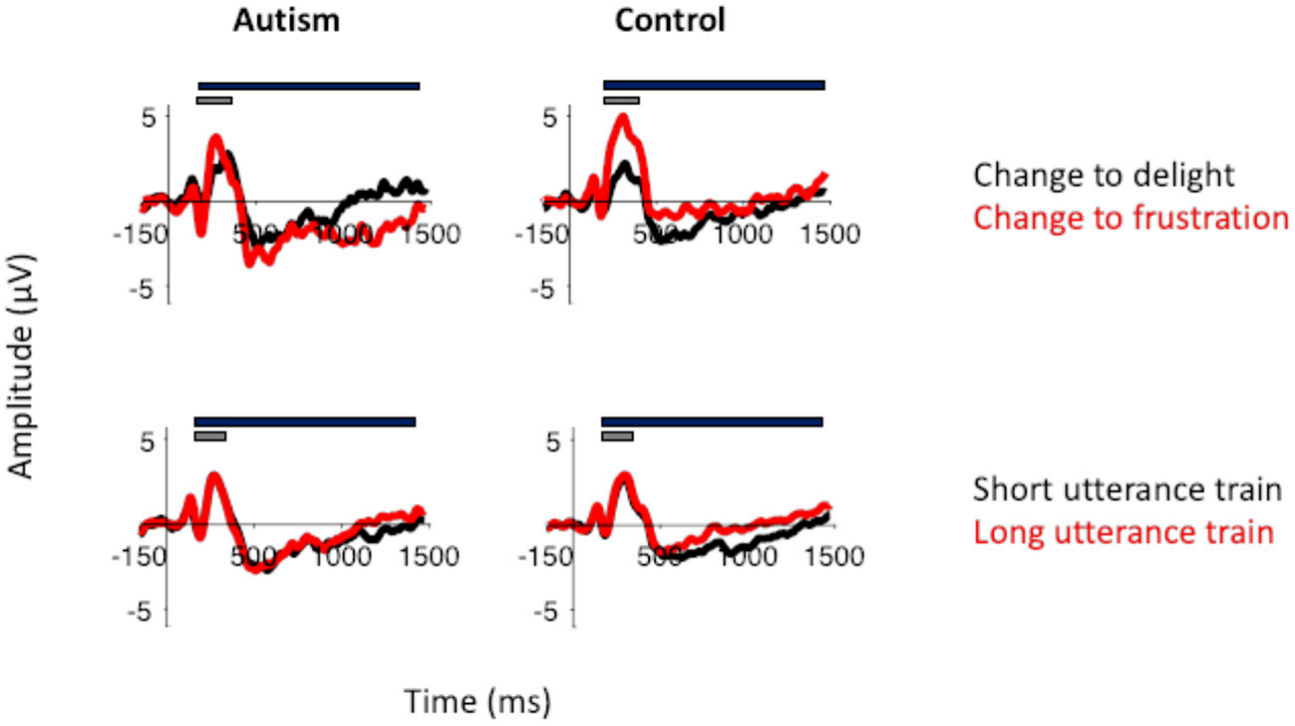
Top row: P3 to the change in utterance to delight (black) and to frustration (red) in autism (left) and neurotypical controls (right). P3 was larger in response to frustration than to delight in controls. This was not the case for the adults with autism. Bottom row: P3 from the change in utterance after a short utterance train (black) and after a long utterance train (red). The gray bar indicates the window used for the peak amplitude analysis. The black bar indicates the window used for the TTV analysis. Both groups showed similar effects of adaptation in their P3 response.

**Figure 8:**
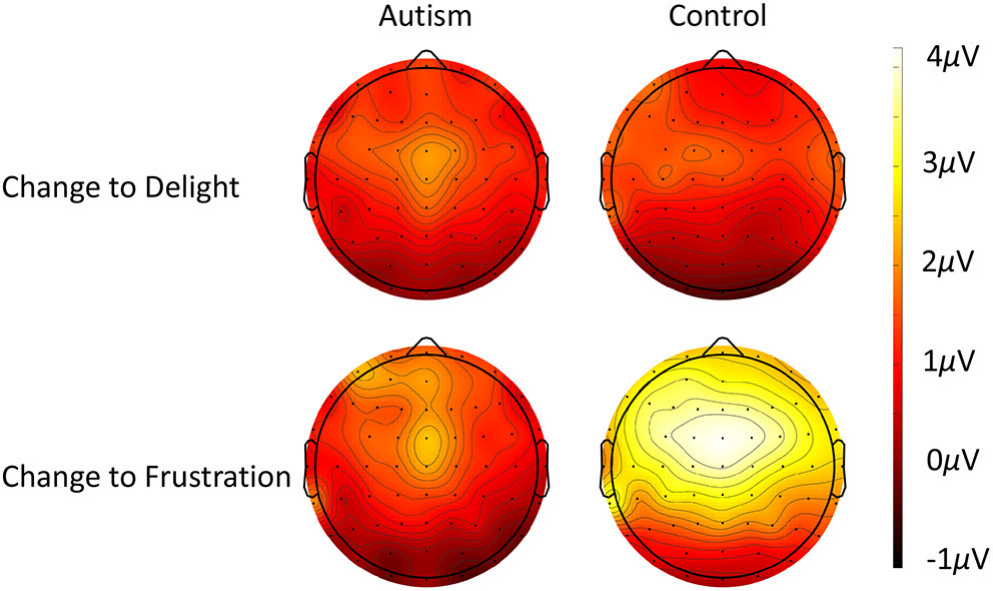
Scalp topography of the P3 response to when the utterance changed from frustration to delight (top row) and when the utterance changed from delight to frustration (bottom row) for autism (left column) and neurotypical controls (right column).

For the P3 response to the changing prosodic utterance, the change from delight to frustration evoked a larger P3 compared to the change from frustration to delight [*F* (2, 51) = 12.60, *p* < 0.001]. While there was no significant difference between groups [*F* (1, 51) = 1.09, *p* = 0.301], there was a significant interaction between group x change in prosody [*F* (1, 51) = 6.71, *p* = 0.012], due to the controls producing a significantly greater P3 to the change from delight to frustration compared to autism [*F* (1, 51) = 4.05, *p* = 0.029; *d* = 0.59; Figure 7]. The P3 response to the change from frustration to delight was similar across the two groups. For all analyses, P3 was largest in FCz electrode (*p* < 0.05; see Figure 8 for topography of P3 response). For the responses to the standard utterances, there was no difference between the delight and frustration utterances [*F* (1, 51) <0.01, *p* = 0.956] or any difference between groups [*F* (1, 51) = 1.09, *p* = 0.301], and so the difference in sensitivity between the autism and control groups was likely due to the change in prosody and unlikely to be due to abnormal responses to all utterances. The waveforms for each electrode location to the change to the delight and to the frustration trials and to the standard utterances for the autism and control groups are shown in Supplementary Figures S10–S13.

**Figure 9:**
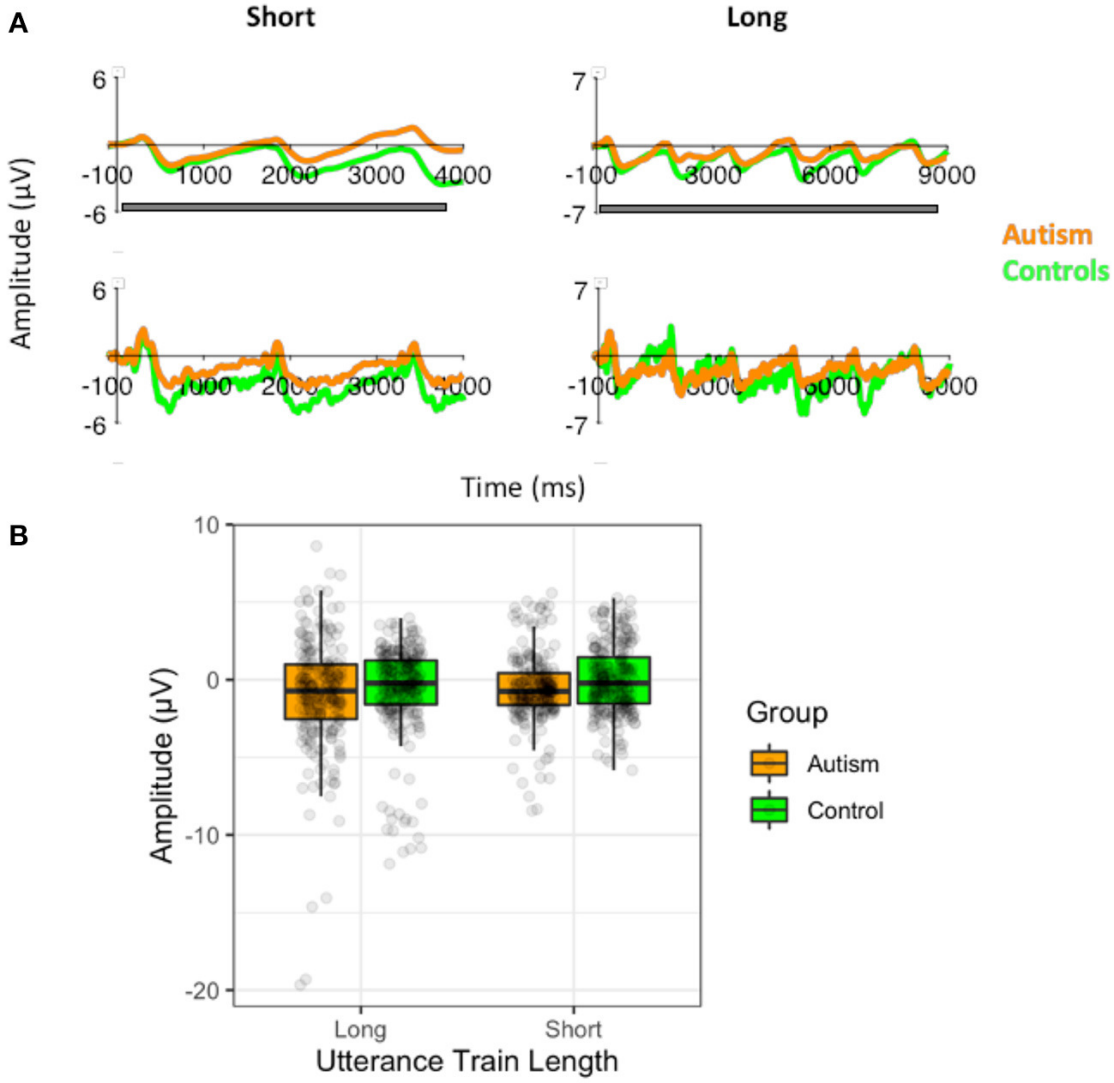
**(A)** Waveforms to the short (3) standard utterance trains and the long standard utterance trains (6) filtered at 1.5Hz for the analysis of the slow-wave (top) and without the filtering (i.e., the original filtered data; bottom), shown separately for individuals with autism and neurotypical controls. Both versions of the filtered waveforms are shown to illustrate the slow-wave that was analyzed and how it arose from the original data used to calculate the P3. The gray bar indicates the window use for the slow-wave analysis. **(B)** Amplitudes of the slow-waves for the long and the short standard utterance trains shown separately for individuals with autism and neurotypical controls.

However, unlike the responses to the pitch stimuli, there was no effect of the number of utterances before the prosody changed on the P3 response [*F* (1, 51) <0.01, *p* = 0.956], no difference between groups [*F* (1, 51) = 1.09, *p* = 0.301], and no significant interaction [*F* (1, 51) = 0.62, *p* = 0.436; Supplementary Figure S11]. There was also no significant effect of the utterance train length on the slow-wave potential [*F* (1, 51) = 0.99, *p* = 0.325], nor was there significant difference between groups [*F* (1, 51) = 1.00, *p* = 0.322], or any interactions with group (Figure 9). Similarly, there were no differences in the standard waveforms to the short and long utterance trains [*F* (1, 51) = 0.097, *p* = 0.331] or any difference between autism and control groups [*F* (1, 51) = 0.04, *p* = 0.842; Supplementary Figure S15].

#### Trial-to-Trial Variability

The TTV in the responses to prosodic utterances was not significantly different between autism and control groups, regardless of whether TTV was calculated across all epochs [*F* (1, 51) = 2.61, *p* = 0.112], or just over the deviant epochs [*F* (1, 51) = 2.06, *p* = 0.158]. Similarly, there was no effect of prosody when analyzing the deviant epochs [*F* (1, 51) = 2.00, *p* = 0.163] or an interaction between group and prosody [*F* (1, 51) = 1.71, *p* = 0.197].

**Figure 10:**
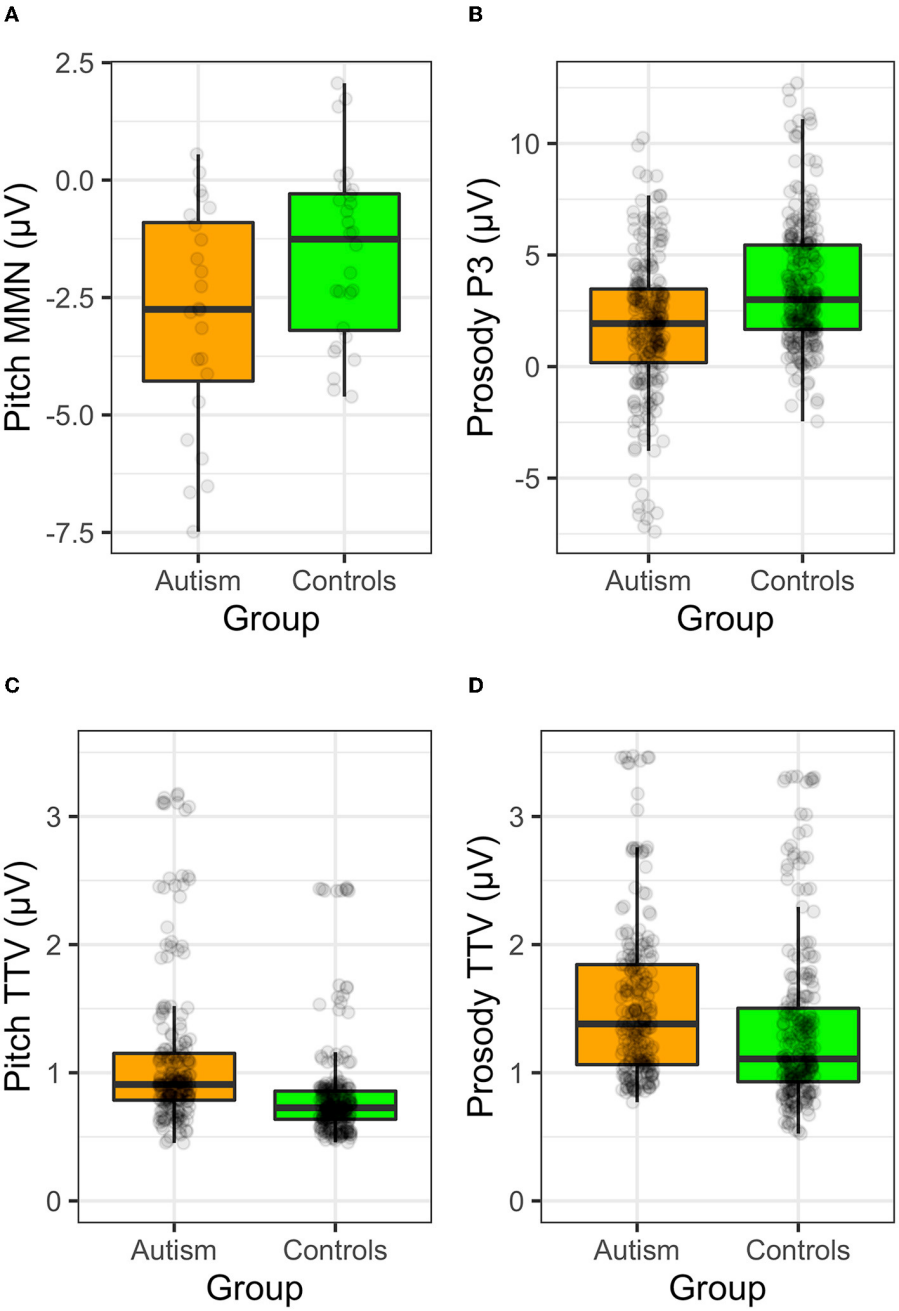
ERP responses to the **(A)** MMN to the long tone trains, **(B)** the P3 to the change to frustration utterance, **(C)** TTV to the pitch tones, and **(D)** TTV to the prosodic utterances, shown separately for individuals with autism and neurotypical controls.

Comparison of ERPs and TTV to the simple tones and the complex prosodic utterances are shown in Figure 10.

#### Reaction Time to Change in Fixation Cross

Once again, to ensure that any group differences in ERPs to the prosodic utterances were not due to differences in attention or fatigue, the response to the change in fixation cross color were analyzed. There was no significant difference between the autism and control groups on their reaction times when responding to the fixation target [*t* (48) = 1.33, *p* = 0.190] or in the number of missed trials [*t* (50) = 0.59, *p* = 0.556] during Experiment 2 either.

#### Relationship Between Prosody Processing and Symptoms

As the P3 response was maximal over FCz (which is typical for deviance detection in complex auditory stimuli) for both groups, ADOS, symptom severity, and IQ scores were correlated with P3 from FCz. There were no significant correlations between symptom scores and P3 responses in the autism group.

#### Exploratory: Examining the Relationship Between Pitch and Prosody Processing Measures

**Figure 11:**
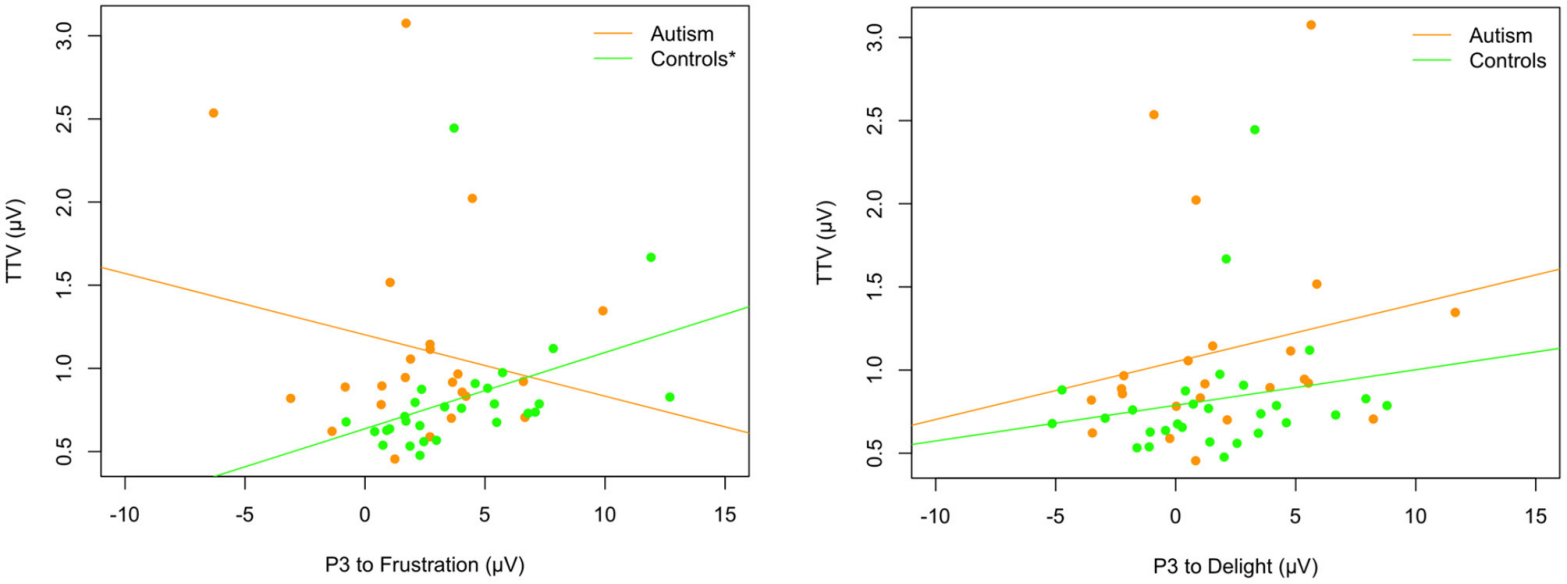
Relationship between trial-to-trial variability and P3 to the change from delight to frustration (left) and from frustration to delight (right) in autism (orange) and neurotypical controls (green). Asterix in legend (left) shows significant correlation for TTV and P3 to frustration in controls only.

To investigate whether there was a relationship between the EEG responses to the pitch stimuli and the prosodic utterance stimuli, the MMN from Fz electrode and the P3 from FCz (where responses were largest) were correlated. When correlating the MMN response after the short and long tone trains with the P3 response to the prosodic utterance stimuli (change from delight or frustration) across the whole sample and separately for each group, none of the correlations were significant (*p* > 0.05; uncorrected for multiple comparisons). However, when P3 responses were correlated with the pitch TTV to all tones, controls who showed greater TTV also tended to exhibit larger P3 responses after the change to the frustration utterance [*r*_*s*_(26) = 0.64, *p* < 0.001] but the control group did not show the same relationship after the change to the delight utterance [*r*_*s*_(26) = 0.31, *p* = 0.104]. Neither comparison was significant for the autism group [frustration: *r*_*s*_(23) = −0.10, *p* = 0.634; delight: *r*_*s*_(23) = 0.23, *p* = 0.286; see Figure 11].

A summary of the results from Experiment 1 and Experiment 2 is shown in Table 2.

**Table 2:** A summary of the ERP findings from the responses to changing pitch (Experiment 1) and changing prosody (Experiment 2).

	**Measures**	**Summary result**
**Experiment 1 Pitch**	MMN after short vs. long train N1 after short vs. long train N1 to standard tones Slow-wave to tone groups MMN to difference in pitch N1 to difference in pitch TTV	Larger MMN in autism after the long tone train in right hemisphere Larger N1 in autism after the long tone train No group difference No group difference No group difference No group difference Greater TTV in autism
**Experiment 2 Prosody**	P3 to deviant utterance	Larger P3 to the change to frustration in controls
	P3 after short vs. long train Slow-wave to utterance groups Standard utterances TTV	No group differences No group differences No group differences No group differences

## Discussion

Sensory abnormalities are characteristic of autism but there are differing and often contradictory reports of how the sensory abnormalities manifest. Here, we assessed neural sensitivity to pitch and to prosody separately but within the same individuals to compare sensitivity at different levels of the auditory processing stream. The adults with autism showed a hyper-sensitivity in their EEG responses to changing pitch but only after long tone trains, suggesting abnormal adaptation compared to the matched neurotypical controls. They also exhibited greater trial-to-trial variability in their responses to all tones. While there were no behavioral differences in tone discrimination between autism and control groups, the autism group was significantly slower.

For prosody processing, the adults with autism showed hypo-sensitivity to the changes in prosodic utterance (specifically from delight to frustration) compared to controls, due to the lack of sensitivity to which emotion is being conveyed. Interestingly, there was no evidence of greater TTV in responses to prosodic utterances in autism. Together, these findings suggest unstable and hyper-sensitive early processing of pitch and reduced neural sensitivity later in the auditory pathway to prosody in autism. It is important to note that the brief behavioral task completed during the study session shows that participants were able to recognize all prosodic utterances and there was no significant difference between groups in their reaction times (see Supplementary Material). Therefore, there is no evidence that the reduced sensitivity in the autism group was due to the slowing or the inability to recognize the utterances.

The behavioral prosodic identification task used in the current study did not detect any group differences. It is possible that this task was not sensitive enough to detect subtle deficits in prosody identification. However, there is some debate as to the robustness of deficits in behavioral prosody recognition in autism in general. Meta-analyses have suggested that the reported deficits in autism may be due to too many options for participants to select from and so are due to methodological constraints rather than a pure deficit in prosody identification (68). Others suggest that the deficits are partially due to the inclusion of individuals with intellectual disability (ID) and that autism without ID individuals tend to only show deficits identifying complex prosodies such as boredom (69). This is despite the robust deficits in facial emotion processing (70). Accounting for sensitivities in P3 responses to simple compared to complex emotions may further elucidate where the neural hypo-sensitivity is maximal.

The abnormal adaptation to the tones in autism was evident in the MMN and did not appear to be related to the slow-wave potential to the standard tones. The slow-wave is theorized to reflect auditory segmentation and is sensitive to abnormal auditory processing in clinical populations such as schizophrenia (51, 52). Therefore, there is little evidence that the hyper-sensitivity in auditory adaptation is due to problems segmenting the auditory scene. Differences in early auditory adaptation can be due to localized to the sensitivity of primary auditory cortex. However, this may be too simple a view. Predictive coding models suggest that there are multiple cortical modules involved in detecting change in the sensory environment (46, 47) and stronger predictive coding in autism would also theoretically generate larger MMNs after the long tone train. From the current data, it is difficult to tease these two theories apart. However, prior studies have shown that predictive coding is in fact weaker in autism compared to controls (71, 72). To directly explore the effect of abnormal adaptation in autism, the length of the tone trains would need to be systematically varied to avoid any possible effects of expectation that may have arisen with only having two tone train lengths.

### Sensory Sensitivity in Autism

Abnormal sensory adaptation and greater TTV is consistent with a growing body of literature suggesting that the hyper-sensitivity and reduced stability are key features of sensory processing in autism that impact behavior. Some variability in the system has been theorized to be helpful when learning statistical regularities in the environment (29, 73, 74). However, too much variability may be detrimental, contributing to the feeling of being overwhelmed by the sensory environment. The greater TTV has been reported previously in auditory processing (30, 31) but also in the visual and somatosensory systems (30–32, 75). What was surprising was that greater TTV in autism was only evident when processing pitch and not when processing prosody. This could be due to instability in early sensory processing that is less prevalent when the signal is processed outside of primary sensory cortex. This also indicates that early sensory information in particular, is unreliable, and when combined with the hyper-sensitivity, could evoke feeling overwhelmed (76, 77). A point to note is that the TTV measured here is based on variability in measures of amplitude; however, the timing of the ERP peaks may also vary and would also impact average ERP responses. Further investigation into the timing of the ERPs and links between sensory hyper-sensitivity and subjective reports of feeling overwhelmed or the impact on social cognition will help elucidate these connections.

The P3 responses to the change in prosody revealed that controls produced larger responses to the frustration utterances compared to the delight utterances. In neurotypical individuals, negative emotions have been shown to elicit larger P3 amplitudes compared to neutral utterances (78, 79), and clinical populations have been shown to be less sensitive to changes in prosody in their P3 response [in schizophrenia, (80)]. Therefore, the lack of sensitivity when shifting between the positive and negative emotions in autism demonstrates reduced sensitivity rather than an overall deficit in encoding prosodic stimuli.

There is a debate as to whether the hypo-sensitivity to stimuli such as prosody in autism is simply due to stimulus complexity. For example, previous studies found that increasing the stimulus complexity spectrally and temporally (timbre) resulted in any advantage in pitch discrimination disappearing (81), and reduced fMRI activation in non-primary auditory cortex in autism compared to controls (82). This suggests deficits in encoding complex stimuli. However, Hudac et al. (23) found that children with autism showed similar slower attenuation in ERP responses to complex novel sounds and to simple infrequent tones, suggesting no such effect of complexity. Similarly, a separate study found that manipulating sound complexity had no impact on ERPs in autism, however, speech did evoke smaller responses in children with autism (83, 84). Similarly, phonemes with consistent pitch features produced large MMNs until the pitch in the phonemes varied and became more speech-like, wherein the MMN was smaller in children with autism compared to controls (5). These findings dispute the theory that auditory complexity is the only feature that impacts later auditory-related processing. Therefore, there may be some specific characteristic of socially-relevant complex stimuli that is impacted in autism (see reference (77) for a review). It should also be noted that most of these findings focused on children with autism, and there may be developmental effects in how prosody is processed.

The exploratory relationship between auditory ERPs and prosody ERPs was not significant, suggesting that the hyper-sensitivity in early auditory processing does not (directly) impact the reduced sensitivity to prosody. However, it is difficult to ascertain whether the non-significant relationship was due to the pitches being used in Experiment 1 not being identical to the fundamental frequencies in the prosodic utterances used in Experiment 2. One way to explore if there is a link between the sensory profiles is to try to reduce the hyper-sensitivity to pitch in autism and the effects on prosody processing. Previous methods focusing on improving sensory processing have shown some improvements in sensory integration abilities [for a review, see (85)]. In schizophrenia, training on discriminating between frequency modulated sweeps has led to cognitive improvements, particularly in verbal learning (86). One of the theorized mechanisms underlying the improvements from sensory training is the training induces neural plasticity which allows for other sensory (and potentially cognitive) domains to be more receptive to learning (87). Therefore, while we have not found a direct relationship between pitch and prosody processing, it is possible that normalizing pitch processing might induce neural plasticity to help improve later auditory processing.

### Limitations

There are several limitations to the investigation we have conducted. The first is that some of the differences between autism and controls in peak ERP responses are small to medium in their effect sizes and are only evident under certain conditions. The sample size is appropriate for these effect sizes, but the exact conditions under which individuals with autism are hyper- or hypo-sensitive needs to be ascertained. For example, are autism less sensitive to changes to all negative emotions or just to frustration? Does the MMN increase in autism linearly with longer tone trains? Systematically varying these conditions could elicit further information on the underlying mechanisms impacting abnormal auditory processing in autism. Second, there are a number of analyses conducted for each experiment and the results were not corrected for multiple comparisons. Therefore, some caution must be taken in case Type 1 error is impacting some of the findings. However, the current results are consistent with prior findings: we replicated the findings of larger MMN in adults with autism (similar to 23), combined with abnormal adaptation compared to controls (23, 25), the abnormal P3 responses to utterances reported previously were in children with autism (5, 37, 38, 88). This is the first study to demonstrate different sensory profiles in the same group of adults with autism. Reducing the alpha to 2.5% would still result in a marginally significant increase in the N1 response to tones in autism compared to controls (Experiment 1) and the reduced sensitivity in the P3 to changes in prosody in autism (Experiment 2). Third, while IQ was assessed in the autism group, it was not for the control group. All the autism group had an IQ > 87, and therefore did not have a marked intellectual disability; however, it is possible that subtle effects of IQ impacted the group differences in ERP responses. It should be noted that there were no significant correlations between ERP responses and IQ measures in the autism group. There is also some evidence that the autism population differs less in their IQ profile than previously thought (88), suggesting that IQ likely does not explain differences on performance in autism. Fourth, the reduced sensitivity to the change to frustration in the autism group may have been impacted by the fact that Experiment 1 always preceded Experiment 2. While we cannot conclusively rule out effects of fatigue, the behavioral responses to the change in fixation color were statistically similar between autism and control groups for both Experiments, and so there is no obvious evidence that fatigue can explain these results. Fifth, we focused on measures of neural sensitivity in our participants to trace the sensitivity to change detection along the auditory hierarchy. There is an assumption that neural sensitivity reflects behavioral sensitivity; however, this is not always the case (89). Our behavioral measures of pitch discrimination and prosody identification (see Supplementary Material) did not identify any differences in performance between autism and control groups, except for slower reaction times in the pitch discrimination task. While these tasks were conducted on a subset of individuals and so there may be some limitation of statistical power, it is more likely that the tasks were not sensitive enough to detect subtle differences in behavioral measures of pitch and prosody sensitivity. Sixth, we biased our autism sample to include only high-functioning adults with ASD. Expanding the sample size to include adults with autism with lower IQ and a larger age range would help identify other factors that are associated with autism and may impact sensory sensitivity.

## Summary

Adults with autism exhibited hyper-sensitivity and greater variability in early auditory processing while also showing reduced sensitivity to changes in prosody. There is no clear relationship between early and later auditory processing suggesting that the different sensory profiles are not related, at least when comparing across autistic individuals. Further investigation into the impact these different sensory profiles have on behavioral functioning will elucidate new targets for behavioral treatment.

## Data Availability Statement

The datasets presented in this study can be found in online repositories. The names of the repository/repositories and Accession Number(s) can be found below: https://osf.io/pnvay/. Further enquiries can be directed to the corresponding author.

## Ethics Statement

The studies involving human participants were reviewed and approved by Institutional Review Board at the University of Nevada, Reno. The patients/participants provided their written informed consent to participate in this study.

## Author Contributions

SH designed the study, analyzed the data, and drafted the paper. PB collected the data and edited the final paper. SE helped with participant recruitment and edited the final paper. DL helped with stimulus design and edited the final paper. DS helped recruit participants and edited the final paper. MB was the mentor on this project and helped write the paper. All authors contributed to the article and approved the submitted version.

## Funding

This project was supported by a NARSAD Young Investigator Grant from the Brain & Behavior Research Foundation (26282) to SH, an R15 AREA award from the National Institute of Mental Health (122935) to SH, an NSF EPSCoR Grant (1632849) on which SH is a Co-Investigator, and a CMU BrainHUB Grant to MB.

## Conflict of Interest

The authors declare that the research was conducted in the absence of any commercial or financial relationships that could be construed as a potential conflict of interest.

## Publisher's Note

All claims expressed in this article are solely those of the authors and do not necessarily represent those of their affiliated organizations, or those of the publisher, the editors and the reviewers. Any product that may be evaluated in this article, or claim that may be made by its manufacturer, is not guaranteed or endorsed by the publisher.
