# Supplementary material for: Hyper-Sensitivity to Pitch and Poorer Prosody Processing in Adults With Autism: An ERP Study
Source: Front Psychiatry. 2022 May 25;13:844830. doi: 10.3389/fpsyt.2022.844830 (PMC9174755; doi:10.3389/fpsyt.2022.844830)
Supplement: Supplementary file 1 [file Data_Sheet_1.PDF]

Hyper-Sensitivity to Pitch is Related to Poorer Prosody Processing in Adults with Autism

Sarah M Haigh[1,2], Pat Brosseau[2], Shaun M Eack[3],
David I Leitman[4], Dean F Salisbury[5], Marlene Behrmann[2,6]

[1]Department of Psychology and Integrative Neuroscience,
University of Nevada, Reno
[2]Department of Psychology, Carnegie Mellon University
[3]School of Social Work, University of Pittsburgh
[4]National Institute of Mental Health
[5]Department of Psychiatry, University of Pittsburgh
[6]Neuroscience Institute, Carnegie Mellon University

The following contains information on the behavioral tasks that were used to verify that both groups could discriminate and identify the sounds, and additional figures to show EEG waveforms for each electrode position used in the analyses.

Experiment 1
Participants

Table 1: Clinical and individual characteristics for all of the participants with autism. The criteria for a diagnosis of autism was a minimum ADOS total score of 10 (combined communication and reciprocal social interaction scores).

Participant	Age	Gender	ADOS Communication	ADOS Social	IQ Full	IQ Verbal	IQ Performance
1	28	F	3	7	92	107	80
2	21	M	8	9	111	102	119
3	44	M	4	10	87	92	85
4	29	M	4	7	104	115	93
5	28	M	4	6	123	120	119
6	26	F	3	11	97	95	98
7	38	F	2	7	123	119	121
8	32	M	2	5	128	122	127
9	41	M	4	4	128	116	134
10	29	M	3	8	123	117	123
11	26	M	4	7	111	100	121
12	24	M	2	5	133	141	133
13	23	M	4	5	127	122	126
14	37	M	4	7	125	118	126
15	19	M	5	9	115	105	122
16	22	M	2	5	107	110	100
17	26	M	3	5	89	98	85
18	31	M	3	4	88	103	77
19	36	F	2	6	120	117	119
20	24	F	4	9	95	86	106
21	41	M	5	5	100	98	103
22	22	M	2	5	99	106	91
23	19	M	5	9	127	121	126
24	19	M	4	4	129	137	116

Behavioral Pitch Discrimination Task
Stimuli

Stimuli were 1046.5Hz (C6), 1062.2Hz, 1077.9Hz, 1108.73Hz (C6 sharp), 1244.51Hz (D6 sharp) tones. Tones were presented in pairs with a 500ms inter-stimulus interval. Half of the pairs were the same tone and the other half contained different tones. For the different tone pairs, the order of whether the highest or the lowest pitch tone was presented first was counterbalanced. Each of the different tone pairs was presented four times. The five temporal frequencies provided ten pitch changes.

Procedure

For all paradigms, a grey screen was presented with a black fixation cross in the center of the screen. The behavioral paradigm was conducted first. For the pitch discrimination task, pairs of tones were presented with 500ms ISI. After both tones were presented, the participant was asked to decide whether the tones had the same pitch or were different. If they sounded the same then the participant pressed the 'S' key and if they were different then they pressed the 'D' key.

Data Analysis

Participants who did not respond at ceiling (greater than 80% likelihood of correctly responding that the tones with the largest pitch difference sounded different) or at floor (less than 20% likelihood or saying that the identical tones sounded different) were removed from analysis (6 ASD and 3 HC) assuming difficulty complying with task instructions. One participant's data did not record correctly. This resulted in responses from 19 ASD and 23 HC being included in the analysis.

To assess whether the groups differed in their ability to discriminate pitch, three points were calculated from the response function from each individual: the top asymptote (at what pitch difference can the participant reliably distinguish between pitch), the midpoint (the threshold at which participants are able to start discriminating between different pitched tones), and the gradient of the function (how reliable is the ability to discriminate between tones – if participants are reliable in their pitch discrimination, then they will exhibit a step-like function where tones either sound the same or they do not). These measures were calculated using a non-linear squares function to ascertain the fit of the data to a sum-of-squares logistic function. All analyses were conducted in R using nls and SSlogis functions and compared using independent-samples t-tests.

The reaction time between the presentation of the second tone and when the participant responded was also recorded and analyzed. A mixed ANOVA was used to assess the effect of group (autism x control) for each level of pitch difference (ten levels).

To verify that the reduced sample size was not the cause of any spurious results, we calculated Bayes Factors using the R package BayesFactor. Final values were computed using Monte Carlo sampling over 50,000 iterations. The Bayes Factors, BF, were then inverted to provide estimates of the probability of the effect occurring under the alternative hypothesis, H1, compared to the null hypothesis, H0, using the effectsize R package. Jeffreys, 1961, interpretation was used.

Results

Overall, there was no significant difference between autism and control groups in the top asymptote (t(37.9)=0.18, p=.862; BF=0.04; strong ev-

idence against under H0; error=12.0), midpoint (t(34.3)=0.37, p=.714; BF=0.05; strong evidence against under H0; error<0.1), or in the gradient of their responses (t(37.2)=0.088, p=.931; BF=0.13; moderate evidence against under H0; error<0.1), suggesting that ASD and HC have similar pitch discrimination thresholds and are similarly reliable in their pitch discrimination (Figure 1A).

For the reaction times, the autism and control groups were compared for each pitch difference (ten in total) in a single mixed-measures ANOVA. The autism group were significantly slower at all pitch differences compared to controls (F(1,40)=5.42, p=.025; d=0.53; BF=1.11; anecdotal evidence in favor under H0; error=2.1; Figure 1B), and reaction times were faster to the large pitch differences (F(9,360)=9.72, p<.001). There was also a significant interaction between pitch difference and group (F(9,360)=2.13, p=.026), due to the difference between autism and control groups being smaller when responding to large pitch differences.

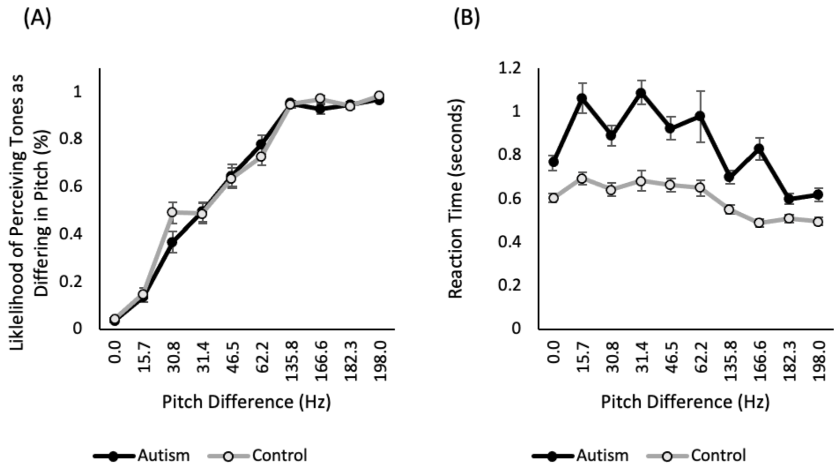

Figure 1: (A) pitch discrimination in the autism and neurotypical control groups. The likelihood of responding that two tones sounded different were similar across the two groups. (B) the reaction times when responding to whether the tones sounded the same or different. The autism group were significantly slower in their response.

These findings verify that perceptually, both groups were able to identify the different pitched tones. Therefore, any differences between the groups in their ERP responses to the tones were due to differences in neural sensitivity and were not due to one group being unable to hear the differences in pitch.

Pitch Waveforms at Each Electrode Site

Waveforms at each electrode to the short and long tone trains in autism and controls showing the subtraction waveform containing the MMN (Figure 2), the N1 response to the deviant tone (Figure 3), and the response to the standard tone (Figure 4). Table 2 shows the summary information for the group x column x length interaction, where the right hemisphere showed the biggest group difference after the long tone train MMN.

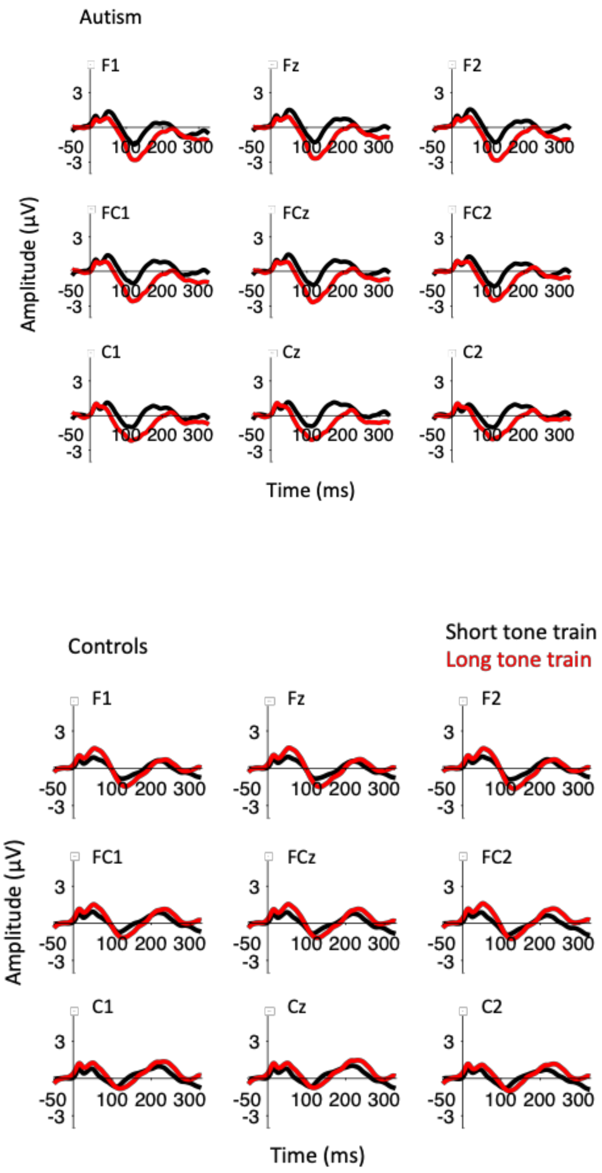

Figure 2: Waveforms to the deviant tone after the short (black) and long (red) tone trains for all electrode sites used in the analysis. (Top) Responses from the adults with autism. (Bottom) responses from the neurotypical controls.

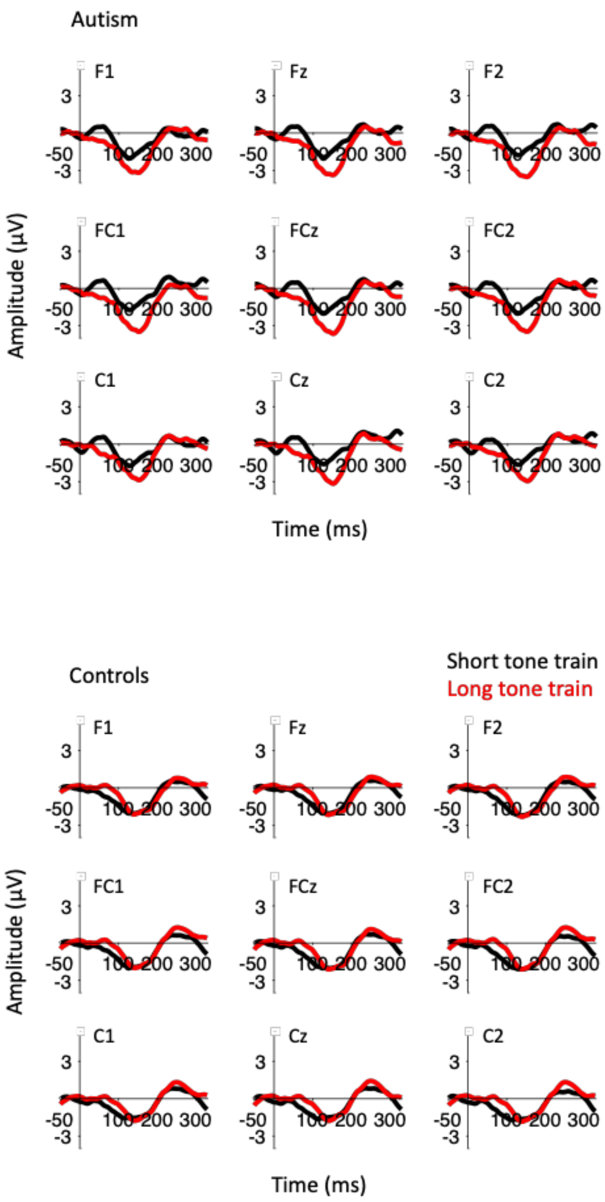

Figure 3: Subtraction to the deviant tone after the short (black) and long (red) tone trains for all electrode sites used in the analysis. (Top) Responses from the adults with autism. (Bottom) responses from the neurotypical controls.

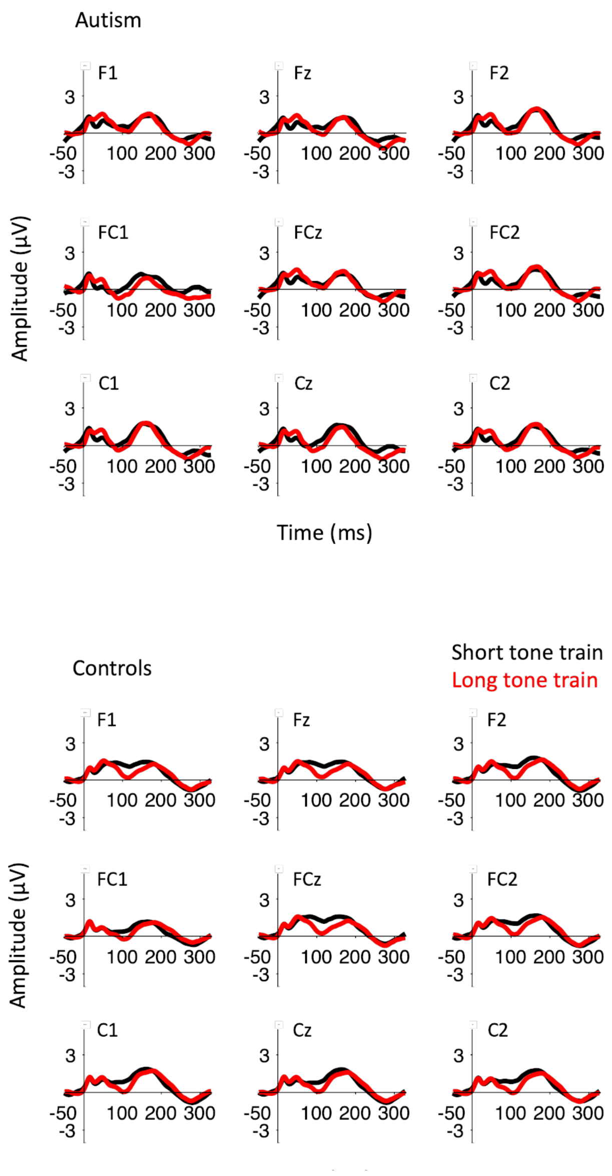

Figure 4: Waveforms to the standard tone during the short (black) and long (red) tone trains for all electrode sites used in the analysis. (Top) Responses from the adults with autism. (Bottom) responses from the neurotypical controls.

Table 2: Short and long MMN means (and SDs) for the left, center, and right columns, shown separately for the autism and control groups.

	**Autism**			**Control**		
	Left	Center	Right	Left	Center	Right
Short MMN	-1.68 (2.29)	-1.78 (2.28)	-1.74 (2.15)	-1.62 (1.78)	-1.86 (1.92)	-1.97 (1.93)
Long MMN	-2.21 (2.49)	-2.64 (2.35)	-2.68 (2.43)	-1.21 (1.92)	-1.40 (1.97)	-1.54 (1.83)

Waveforms at each electrode to the small, medium, and large pitch differences in autism and controls showing the N1 response to the deviant tone (Figure 5), the subtraction waveform containing the MMN (Figure 6), and the response to the standard tone (Figure 7).

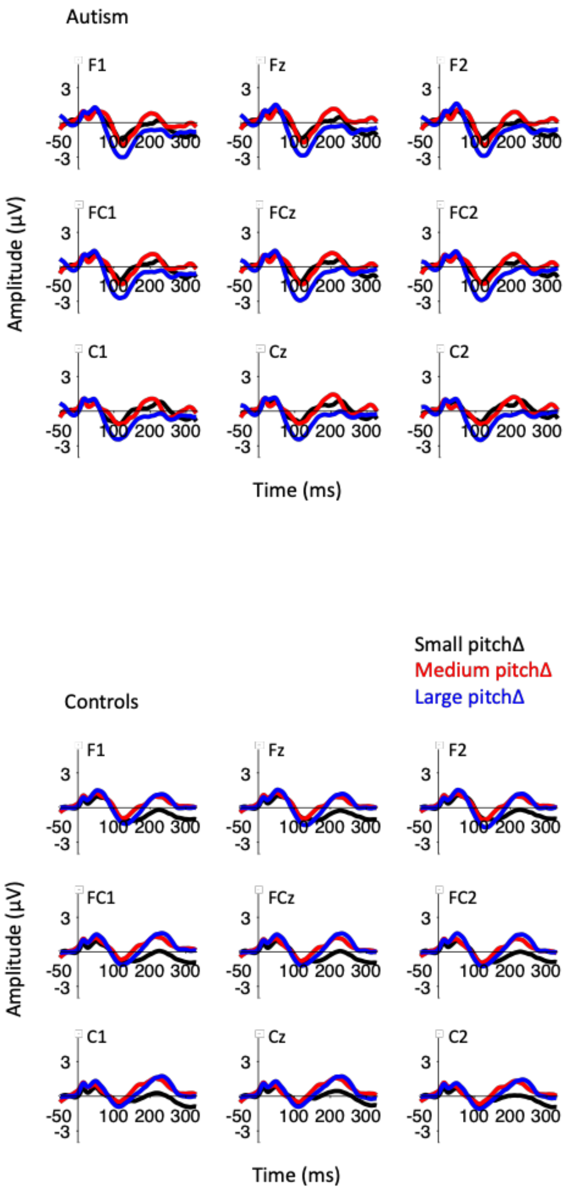

Figure 5: Waveforms to the small (black), medium (red), and large (blue) pitch deviants for all electrode sites used in the analysis. (Top) Responses from the adults with autism. (Bottom) responses from the neurotypical controls.

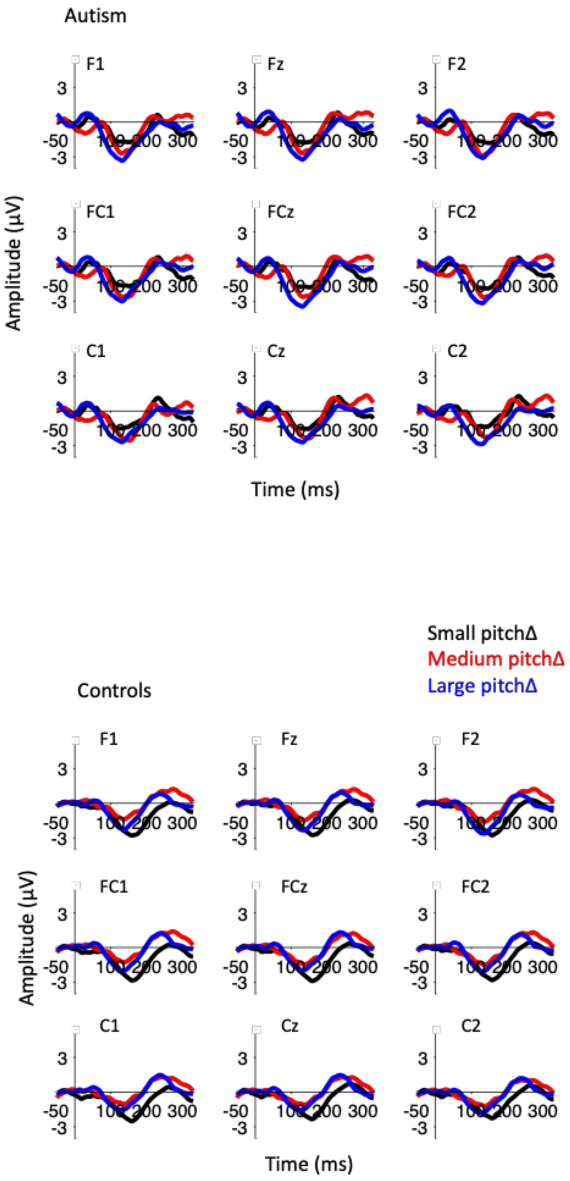

Figure 6: Subtraction waveforms to the small (black), medium (red), and large (blue) pitch deviants for all electrode sites used in the analysis. (Top) Responses from the adults with autism. (Bottom) responses from the neurotypical controls.

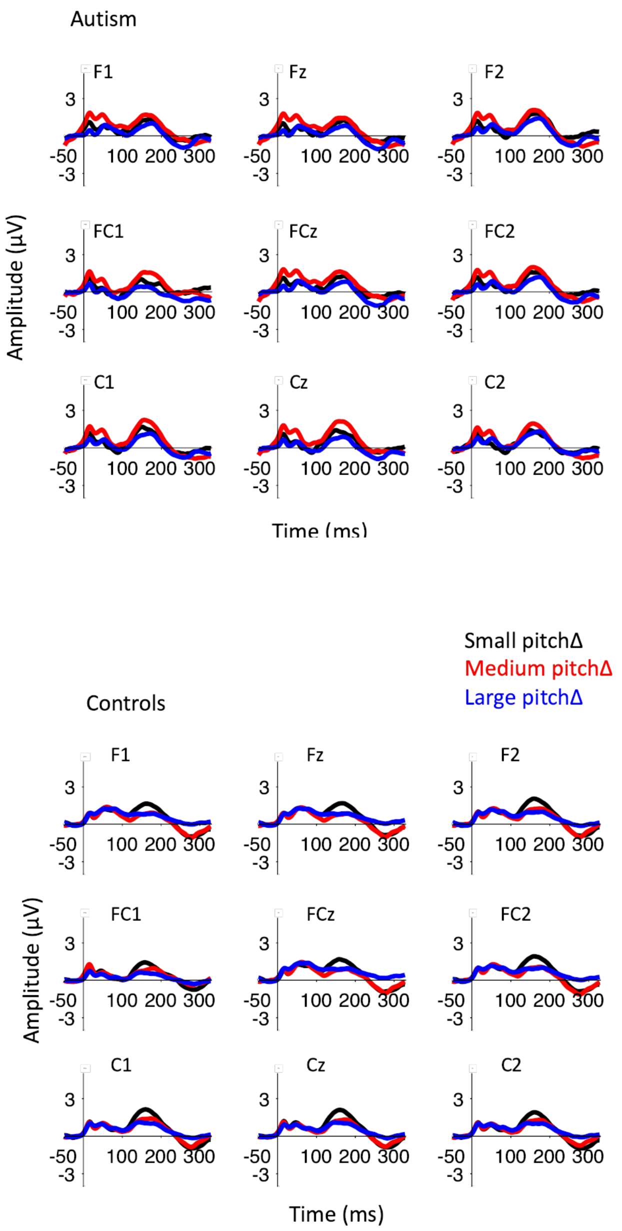

Figure 7: Waveforms to the standard tones that preceded the small (black), medium (red), and large (blue) pitch deviants for all electrode sites used in the analysis. (Top) Responses from the adults with autism. (Bottom) responses from the neurotypical controls.

Experiment 2

Behavioral Prosodic Identification Task

Stimuli

Four examples of laughter, frustration, disgust, delight, and surprise taken from Simon-Thomas et al. (2009) were presented for 2s. All utterances were nonverbal. For each utterance, two examples were from male speakers, and two examples were from female speakers.

Procedure

For the behavioral prosody identification task, an utterance was presented, and the participant had to indicate which emotion the utterance sounded like by responding to the corresponding key on the keyboard. The options were Laughter (press the number 1 key), Frustration (number 2 key), Disgust (number 3 key), Delight (number 4 key), or Surprise (number 5 key).

Data Analysis

The probability of correctly identifying the prosody was calculated for each category of prosodic utterance (laughter, frustration, disgust, delight, and surprise). A mixed-measures ANOVA was used to compare group (autism x control) on each prosody (5 levels). Due to technical difficulties, only 14 ASD and 19 HC's prosodic utterance identification data were collected and analyzed.

Once again, to verify that the reduced sample size was not the cause of any of the group effects, we calculated Bayes Factors using the R package BayesFactor, computed using Monte Carlo sampling over 50,000 iterations, interpreted using Jeffreys, 1961.

Results

Overall, the two positive emotions (delight and laughter) were more likely to be identified correctly compared to the negative emotions. Specifically, delight was more likely to be identified correctly compared to frustration and surprise, whereas laughter was also more likely to be correctly identified compared to disgust ($F(4,124)=17.57$, $p<.001$; Bonferroni corrected pairwise t-tests, $p<.05$). However, there was no significant difference in accuracy between the autism and control groups ($F(1,31)=1.14$, $p=.294$; $BF=0.86$; anecdotal evidence against under the H0; error=0.8) and no significant interaction ($F(4,124)=0.56$, $p=.696$; Figure 8A).

Laughter was identified faster than all other emotions, whereas surprise was identified the slowest ($F(4,124)=15.99$, $p<.001$; Bonferroni corrected pairwise t-tests, $p<.05$). There was still no difference between the autism and control groups ($F(1,31)=0.03$, $p=.868$; $BF=0.90$; anecdotal evidence against under the H0; error=1.0) and no significant interaction ($F(4,124)=0.30$, $p=.877$; Figure 8B).

These findings verify that perceptually, both groups were able to identify the different prosodic utterances, including the ones used in the ERP study. Therefore, any differences between the groups in their ERP responses to the utterances were due to differences in neural sensitivity and were not due to one group being unable to identify or were slower at identifying the different utterances.

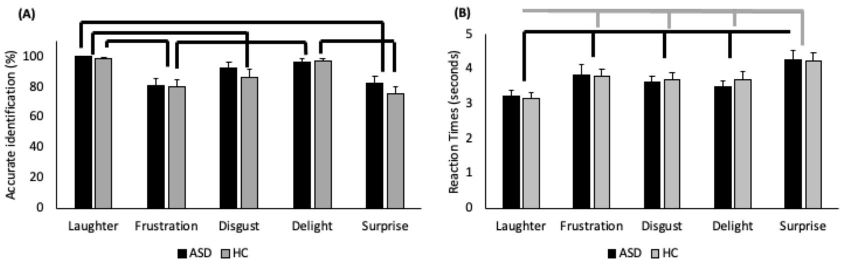

Figure 8: (A) Accuracy at identifying the prosodic utterance and (B) reaction times for each prosody shown separately for autism and neurotypical control groups. Significant pairwise comparisons highlighted.

Prosody Spectrograms

Spectrograms of the stimuli used in the roving prosody EEG paradigm

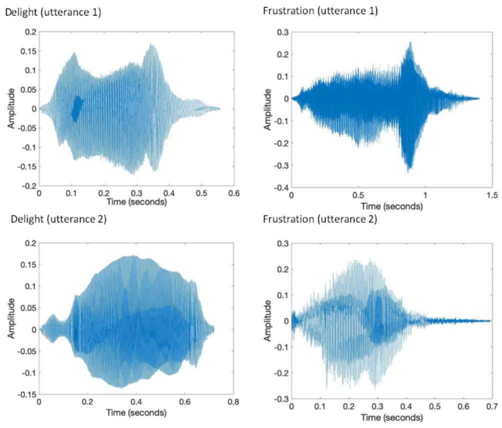

Figure 9: Spectrograms of the prosodic utterances used in the roving prosody EEG paradigm.

Prosody Waveforms at Each Electrode Site

Waveforms at each electrode to the change in prosody from frustration to delight and from delight to frustration (Figure 10) and after the short and long utterance trains (Figure 11) in autism and controls showing the P3 response. The waveforms to the standard delight and frustration utterances (Figure 12), and the short and long utterance trains (Figure 13) in autism and controls for all electrode sites. The timing of the second positivity to more consistent with a P2 and is 50ms earlier than the peak analyzed for the P3 in the deviant waveforms.

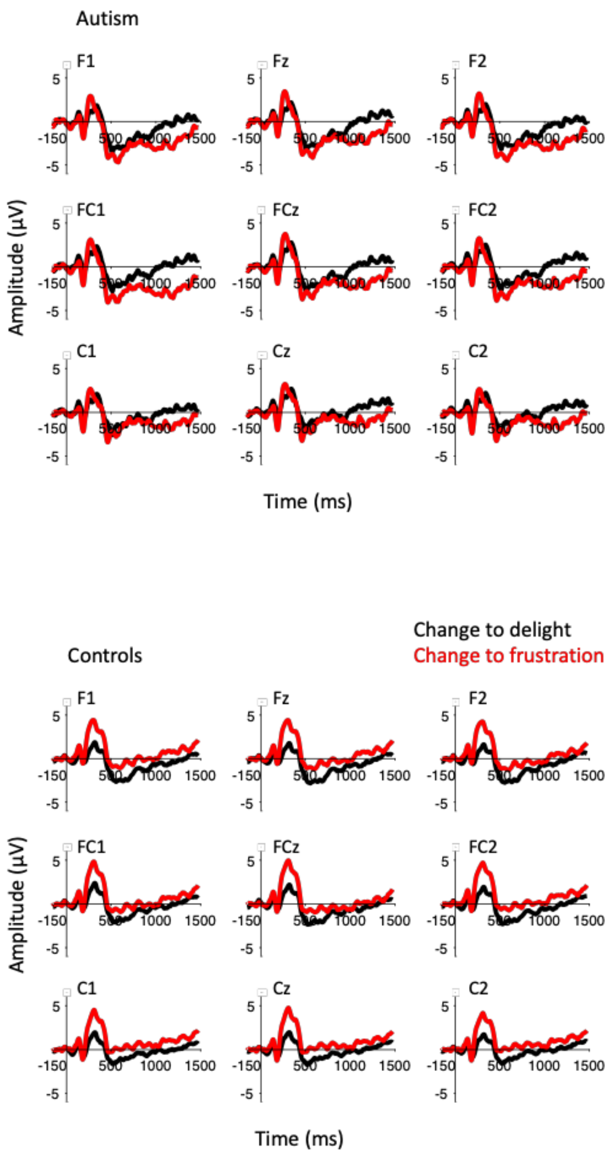

Figure 10: Waveforms to the change to delight (black) and change to frustration (red) prosodic deviants for all electrode sites used in the analysis. (Top) Responses from the adults with autism. (Bottom) responses from the neurotypical controls.

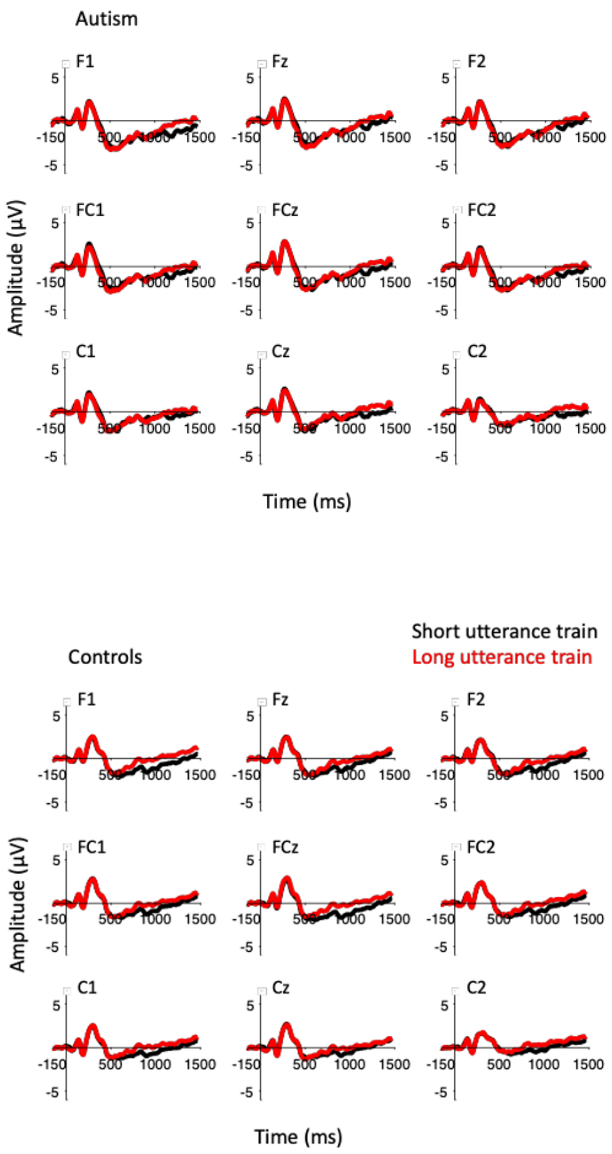

Figure 11: Waveforms to the change in prosody after the short utterance train (black) and the long utterance train (red) for all electrode sites used in the analysis. (Top) Responses from the adults with autism. (Bottom) responses from the neurotypical controls.

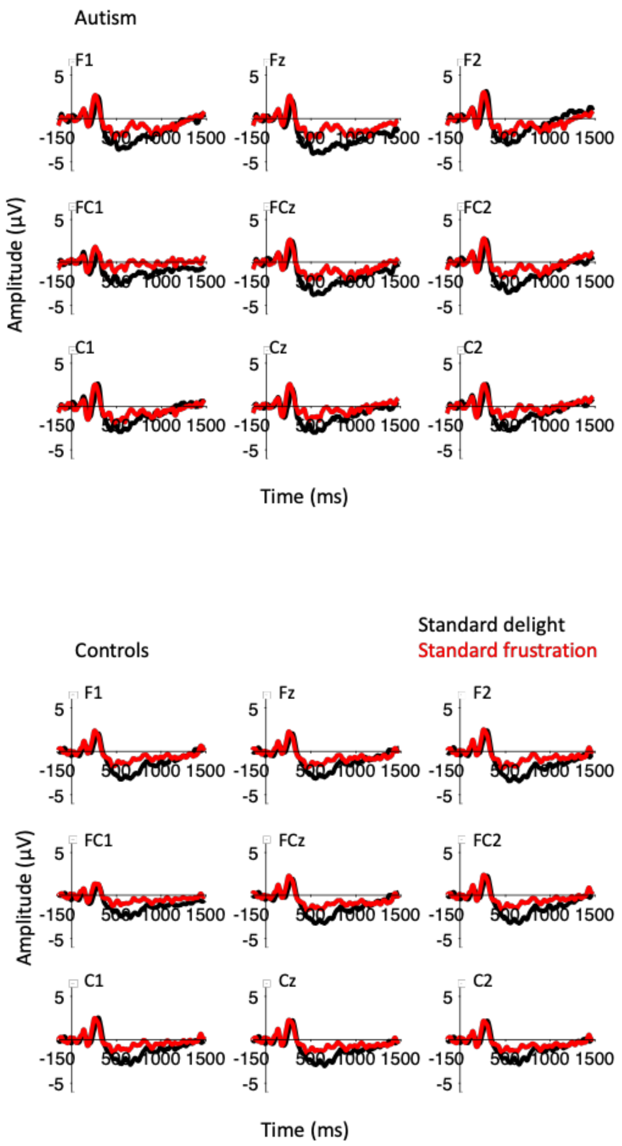

Figure 12: Waveforms to the standard prosodic utterances from the delight standards (black) and the frustration standards (red) for all electrode sites used in the analysis. (Top) Responses from the adults with autism. (Bottom) responses from the neurotypical controls.

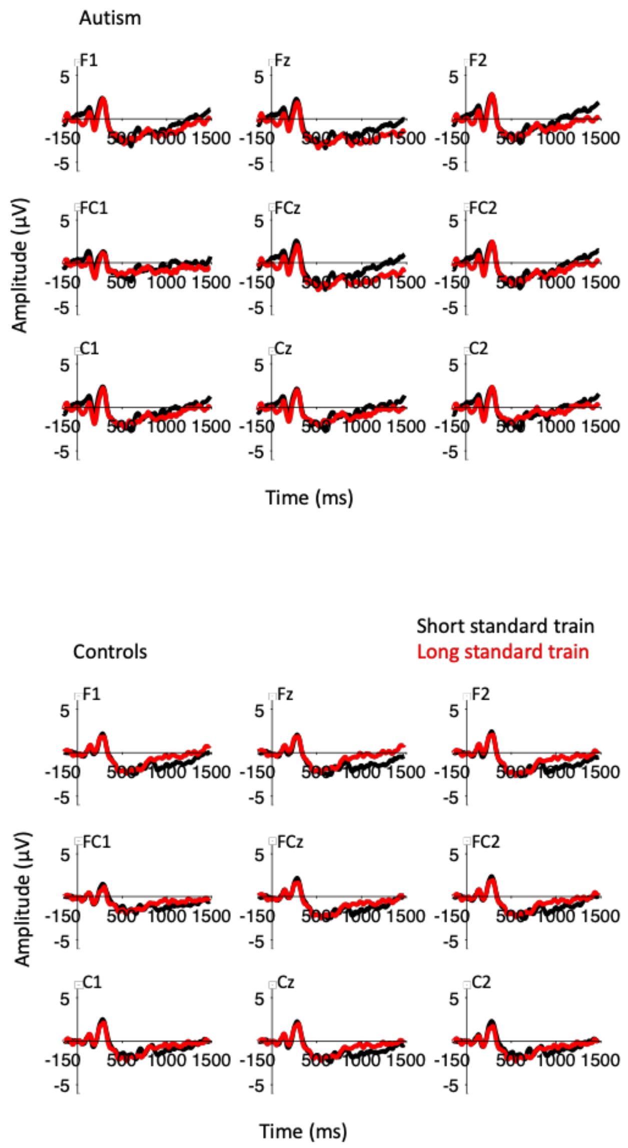

Figure 13: Waveforms to the standard prosodic utterances from the short train standards (black) and the long train standards (red) for all electrode sites used in the analysis. (Top) Responses from the adults with autism. (Bottom) responses from the neurotypical controls.

Supplemental References

Jeffreys, H. (1961). Theory of probability. Oxford, England:Clarendon.
